# Exploring Tyramine's Role in the Formation of Supramolecular Adducts with Nonsteroidal Anti‐Inflammatory Drugs

**DOI:** 10.1002/chem.202500080

**Published:** 2025-04-03

**Authors:** Chiara Rosso, Rebecca Birolo, Angelo Gallo, William T. Franks, Emanuele Priola, Michele R. Chierotti, Roberto Gobetto

**Affiliations:** ^1^ Department of Chemistry and NIS Center University of Turin Via P. Giuria 7 Turin 10125 Italy; ^2^ Department of Physics University of Warwick Coventry, CV4 7AL West Midlands England

**Keywords:** Crystal engineering, Pharmaceuticals, Solid‐state NMR, Solubility, Supramolecular adducts

## Abstract

In pharmaceutical crystal engineering, salification and co‐crystallization are well‐established strategies to enhance the physicochemical properties of nonsteroidal anti‐inflammatory drugs (NSAIDs), which typically exhibit low aqueous solubility. This study introduces three new multicomponent crystalline systems of NSAIDs (*S*‐naproxen, flurbiprofen, and ketoprofen) with the co‐former tyramine, designed using knowledge‐based methods. Additionally, a new polymorph of the diflunisal–tyramine system, synthesized via mechanochemical techniques, is reported. The new multicomponent systems were thoroughly characterized using solid‐state NMR and single‐crystal X‐ray diffraction. Aqueous solubility tests conducted through solution ^1^H NMR experiments revealed increased equilibrium solubility for all samples, highlighting the efficacy of crystal engineering in modulating the physicochemical properties of drugs.

## Graphical abstract

An *a* *priori* design approach identified tyramine as an ideal co‐former for obtaining new supramolecular adducts with nonsteroidal anti‐inflammatory drugs, speeding up the co‐crystallization process. Three novel adducts and a new polymorphic form were synthesized and thoroughly characterized by solid‐state NMR and single‐crystal XRD. The resulting adducts showed an improved equilibrium aqueous solubility.

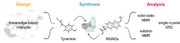

## 1 Introduction

Nonsteroidal anti‐inflammatory drugs (NSAIDs) are widely prescribed and consumed worldwide. Millions of prescriptions and over‐the‐counter transactions occur daily, thanks to their anti‐inflammatory, analgesic, antipyretic, and antiplatelet properties.^[^ ^1^ ^]^ Despite their benefits, NSAIDs typically belong to Class II of the Biopharmaceutical Classification System (BCS) due to their poor water solubility. This inherent limitation reduces their bioavailability, requiring higher doses to achieve good therapeutic efficiency. To address this challenge, various formulation strategies, such as solid dispersions, microemulsions, nanoparticles, salts, or co‐crystals formation have been widely explored.^[^ ^2^ ^]^ Among these, crystal engineering leverages salification and co‐crystallization to modulate the physicochemical properties of drugs by incorporating one or more pharmaceutically acceptable organic molecules, known as co‐formers, into the crystal lattice.^[^ ^3^, ^4^, ^5^, ^6^ ^]^ As a consequence, thousands of multicomponent crystal structures have been deposited and several drugs have been marketed in these solid forms (e.g., ketoprofen–lysine,^[^ ^7^, ^8^ ^]^ ibuprofen–lysine,^[^ ^9^ ^]^ and diclofenac–proline^[^ ^10^ ^]^). The driving force of co‐crystallization lies in the formation of supramolecular synthons between the two molecules within the unit cell, generally involving the strongest basic and acidic groups.^[^ ^11^, ^12^ ^]^ Molecular salts differ from co‐crystals in the hydrogen position along the hydrogen bond, i.e., whether a proton transfer occurs or not.^[^ ^13^ ^]^ This could be predicted from the “Δp*K_a_* rule”^[^ ^14^, ^15^, ^16^ ^]^ and has to be experimentally assessed by a comprehensive characterization of the samples.

**Figure 1:**
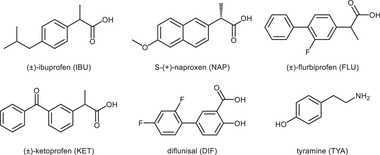
Molecular structures of IBU, NAP, FLU, KET, DIF, and TYA.

In this work, we leverage structure‐based predictive tools to identify a promising co‐former for synthesizing molecular adducts with several NSAIDs: (±)‐ibuprofen (**IBU**), (±)‐flurbiprofen (**FLU**), and (±)‐ketoprofen (**KET**), commercially available in racemic form, *S*‐(+)‐naproxen (**NAP**), marketed as a pure enantiomer, and diflunisal (**DIF**) (Figure 1).

Full interaction maps (FIMs), motif searches, and molecular complementarity (MC) analysis highlight the propensity of the carboxylic acid group of NSAIDs to interact with amine and hydroxyl groups.^[^ ^17^, ^18^ ^]^ We selected tyramine (**TYA**, Figure 1), a derivative of the amino acid tyrosine, as a promising co‐former, considering its chemical structure and its co‐administrability with drugs,^[^ ^19^, ^20^, ^21^ ^]^ as it is listed in the Substances Added to Food (formerly EAFUS) database. Mechanochemical techniques were employed to achieve co‐crystallization, and the supramolecular interaction motifs in the obtained compounds were investigated by combining solid‐state NMR (SSNMR) with powder (PXRD) and single‐crystal (SCXRD) X‐ray diffraction information. Moreover, improvements in the water solubility were investigated via solution NMR solubility tests.

This study investigates key aspects of pharmaceutical crystal engineering through the synthesis of novel supramolecular adducts with NSAIDs. It emphasizes the benefits of rational design in accelerating research timelines, the growing use of green synthetic techniques, and the overarching goal of modulating the physicochemical and, consequently, the pharmacological properties of a target drug. Furthermore, the versatility and broad applicability of both solid‐state and solution NMR in drug discovery and development are showcased, underscoring its pivotal role in the investigation of solid forms, as well as in the analysis of the physicochemical properties, such as solubility.^[^ ^22^, ^23^ ^]^ Therefore, the present study aims to contribute to the establishment of a standardized procedure in the production of new multicomponent crystalline systems, from the design to the analysis.

## 2 Results and Discussion

### 2.1 Rational Design of NSAIDs–TYA Supramolecular Adducts

**Figure 2:**
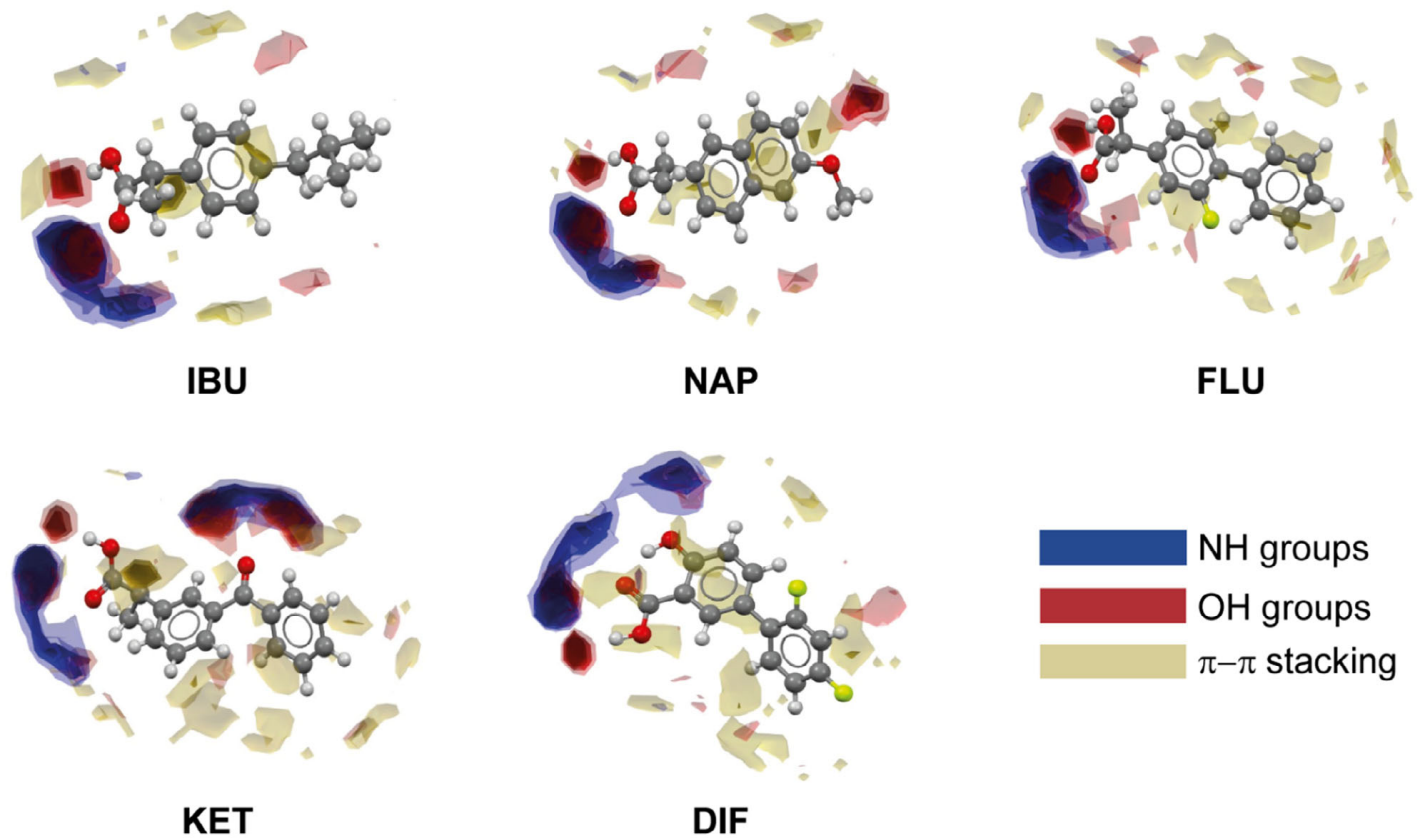
Full interaction maps of IBU, NAP, FLU, KET, and DIF. Colored regions represent the probability of forming π–π stacking interactions (beige) and interactions with NH (blue), or OH (red) groups.

The vast space of chemical compounds suitable for supramolecular adducts formation of NSAIDs has been explored by leveraging the supramolecular synthon theory and knowledge‐based methods to identify a promising co‐former for experimental trials.^[^ ^17^, ^24^, ^25^ ^]^ All investigated NSAIDs are characterized by phenylpropionic (**IBU**, **NAP**, **FLU**, and **KET**) or benzoic (**DIF**) moieties, both having a carboxylic acid group poised for supramolecular interactions, as corroborated by FIMs generated by mercury (Figure 2). The carboxyl group is the molecular region with the greatest propensity for supramolecular interactions: the carbonyl part acts as a hydrogen bond acceptor preferentially with NH or OH donor groups, while the OH portion tends to interact with other hydroxyl groups. The 3D maps also highlight the propensity of the aromatic rings of NSAIDs to establish weak π–π interactions. The occurrence of four possible hydrogen bond interactions (labelled **a**, **b**, **c**, and **d** in Table 1) in the formation of any type of synthon involving COOH with aliphatic amines or phenolic hydroxyl groups was investigated through the motif search tool on Mercury (Supporting Information, paragraph 1.1). The amine group exhibited a high probability of interacting via hydrogen bonding with the C═O moiety (42.5%) of the carboxyl group, more than with the hydroxyl moiety (16.2%). In contrast, the phenolic hydroxyl group showed a lower likelihood of interacting with the COOH group (15.1% with the C═O moiety and 5.5% with the OH one). However, given the remarkable frequency of the interactions **a**, **c**, and **d**, **TYA** was selected as an ideal co‐former for the adduct formation due to its chemical structure, which contains both the functional groups considered.

**Table 1:** Motif search analysis for COOH groups with aliphatic NH_2_ and phenolic OH (on CSD 5.45).

Interaction	# Structure CSD	% Frequency	Total Structures
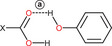	465	15.1	3075
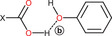	170	5.5	
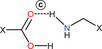	919	42.3	2171
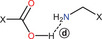	352	16.2	

Three molecular salts of NSAIDs with **TYA** (**IBU‐TYA**,^[^ ^26^ ^]^ **DIF‐TYA**,^[^ ^27^ ^]^ named here **DIF‐TYA Form I**, and **NFA‐TYA**,^[^ ^19^ ^]^ where **NFA** is niflumic acid) were already reported, corroborating the hypothesis that **TYA** is a promising co‐former for achieving new supramolecular adducts with NSAIDs.

For further validation, the MC analysis^[^ ^18^ ^]^ was also performed for **NAP–TYA**, **FLU–TYA**, and **KET–TYA**. This tool reports the percentage probability of co‐crystal formation, given by the number of different API conformations that are complementary with the co‐former, assessing shape, size, and interaction sites of the two molecules. Ten conformations for **NAP**, **FLU**, and **KET** were evaluated. The results suggest a high probability of forming multicomponent crystals with **FLU** and **NAP** (100%), while **KET–TYA** appears unpromising for co‐crystallization (40%). Nevertheless, driven by the structural analogy of this class of compounds, the **KET–TYA** system was also tested.

### 2.2 Experimental Results

Three novel supramolecular adducts of **TYA** with **NAP**, **FLU**, and **KET** were obtained by kneading.^[^ ^28^, ^29^ ^]^ While **IBU‐TYA** and **DIF‐TYA** molecular salts were already obtained by solvent‐based methods,^[^ ^26^, ^27^ ^]^ a greener mechanochemical approach yielded a new **DIF‐TYA** crystal form, named **Form II**. The powder samples were screened by FTIR–ATR (Supporting Information, Figures S3–S6) and PXRD analysis (Supporting Information, Figures S13–S16) to check the formation of new and pure crystalline phases.^[^ ^30^ ^]^ The neutral or ionic nature of the adducts predicted by the “Δp*K_a_* rule”^[^ ^15^, ^16^ ^]^ (Table 2) was investigated using ^13^C CPMAS SSNMR experiments in combination with SCXRD analysis or 2D SSNMR experiments, when suitable single crystals were not achieved (**KET–TYA**) or when the proton location in the supramolecular interaction was unclear (**DIF‐TYA Form II**).

**Table 2:** p*K_a_* of the co‐former TYA and the APIs and Δp*K_a_* [= p*K_a_* (base) – p*K_a_* (acid)] values for the adducts with the predicted outcome (salt/co‐crystal).

Compound	Acid/Base	p*K_a_*	Δp*K_a_*	Predicted Salt/Co‐Crystal
TYA	Base	9.66^[^ ^a^ ^]^	–	–
IBU	Acid	4.91^[^ ^a^ ^]^	4.75	Salt
NAP	Acid	4.15^[^ ^a^ ^]^	5.51	Salt
FLU	Acid	4.22^[^ ^a^ ^]^	5.44	Salt
KET	Acid	4.00^[^ ^a^ ^]^	5.66	Salt
DIF	Acid	3.30^[^ ^31^ ^]^	6.36	Salt

^[a]^ PubChem.

### 2.3 Crystal Structure Analysis

The crystal structures of **NAP–TYA**, **FLU–TYA**, and **DIF‐TYA Form II** (Supporting Information, Figures S20, S21, and S22) reveal the formation of the heterosynthon between the carboxylic acid group of the APIs and the aliphatic amine group of **TYA**, in agreement with prior observations in **IBU‐TYA**,^[^ ^26^ ^]^ **DIF‐TYA Form I**,^[^ ^27^ ^]^ and **NFA‐TYA**.^[^ ^19^ ^]^ This interaction is consistent with the predictions from FIMs and motif searches, confirming the robustness of the ‐COOH···NH_2_ aliphatic synthons. Moreover, in the new structures, other hydrogen bonds between phenolic hydroxyl and carbonyl groups were observed. These interactions contribute to the formation of two‐dimensional hydrogen bond networks and highlight the advantage of selecting a co‐former that contains both aliphatic NH_2_ and phenolic OH moieties.

The crystallographic analysis of **NAP–TYA**, **FLU–TYA**, and **DIF–TYA Form II** suggests the formation of molecular salts, with a stoichiometry of 1:1, via proton transfer from the carboxylic acid group of the API to the aliphatic amine of **TYA**. The formation of carboxylate anions in **NAP–TYA** and **FLU–TYA** is confirmed by the similarity of the C─O bond lengths reported in Table 3. **DIF‐TYA Form II**, instead, shows a larger C─O bond length difference (∼ 0.05 Å), placing its ionic or neutral nature in question. Indeed, it was shown that carboxylate salts typically exhibit C─O bond length differences within 0.03 Å, while for neutral carboxylic acid these lengths can differ by more than 0.08 Å.^[^ ^32^ ^]^

**Table 3:** Bond distances C─O (Å) of the APIs carboxylate groups in the adducts NAP‐TYA, FLU‐TYA, and DIF‐TYA Form II.

	C─O (Å)	C─O (Å)	Δ(C─O) (Å)
**NAP–TYA**	1.248	1.250	0.002
**FLU–TYA**	1.248	1.253	0.005
**DIF‐TYA Form II**	1.235	1.281	0.046

**Figure 3:**
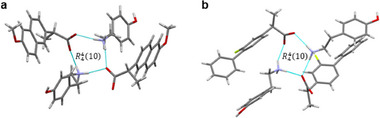
(a) NAP‐TYA and (b) FLU‐TYA $R_{4}^{4}\left(10\right)$ ring motifs.

To clarify the neutral or ionic nature, the adduct was further investigated by advanced SSNMR experiments (see below). In any case, the SCXRD analysis offered insights into the crystalline packing of the system. All the details on the crystal structures of the three adducts and the hydrogen bond patterns into them are reported in Paragraph 3 in the Supporting Information. However, two distinct aspects of these structures should be underlined: (*i*) the formation of the same $R_{4}^{4}\left(10\right)$ ring motif in both **NAP–TYA** and **FLU–TYA** (Figure 3), also observed in **IBU–TYA**, indicating a structural trend across the phenyl propionic NSAIDs‐**TYA** adducts, and (*ii*) the discovery of a new polymorph for **DIF‐TYA**, which present a 2D hydrogen bond network absent in the previous form, **DIF‐TYA Form I**.

### 2.4 Solid‐State NMR

^13^C CPMAS SSNMR was employed to verify the formation of the multicomponent crystalline systems and to investigate their protonation states. In general, the chemical shift of the carboxylic carbon signal provides valuable insights into hydrogen bond interactions, being affected by the proton location along the hydrogen bond axis. This signal typically shifts to higher frequencies in the presence of hydrogen bonding interactions, dimerization through homosynthon formation, or carboxylate formation.^[^ ^32^, ^33^, ^34^, ^35^ ^]^ Therefore, the variation in the chemical shift depends on the system under investigation and its specific hydrogen bond network. Furthermore, SSNMR spectra and NMR parameters can provide other essential information on the number of independent molecules in the unit cell (Z′), the degree of crystallinity, and the presence of disorder.^[^ ^32^, ^36^, ^37^ ^]^

**Figure 4:**
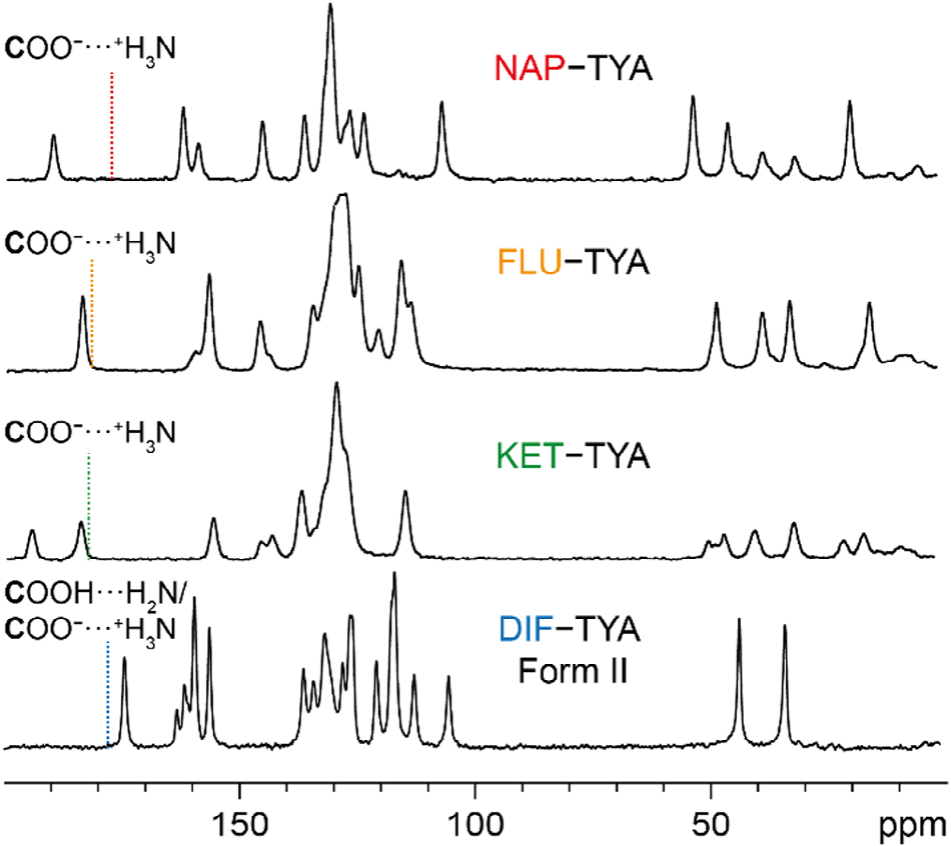
^13^C (100.63 MHz) CPMAS spectra of NAP–TYA, FLU–TYA, and KET–TYA acquired with a spinning speed of 12 kHz at room temperature and ^13^C (150.91 MHz) CPMAS spectrum of DIF‐TYA Form II acquired with a spinning speed of 20 kHz at room temperature. The COOH signals of the pure APIs are represented by the colored dashed lines in each spectrum to highlight the shift undergone upon adduct formation.

The ^13^C CPMAS spectra for each adduct are shown in Figure 4. A detailed comparison with the spectra of the starting materials is reported in the Supporting Information (Figures S23–S26 and Tables S2–S5).

For **NAP‐TYA** and **FLU‐TYA**, the ^13^C CPMAS SSNMR analyses confirm the crystallographic results. These spectra show consistent shifts in all the ^13^C signals, indicating the formation of new crystalline phases characterized by a good degree of crystallinity (full width at half maximum in the range of 120–180 Hz). The number of signals in each spectrum confirms the presence of one independent molecule of **TYA** and one of API in the unit cell and, therefore, a stoichiometry of 1:1. The high‐frequency shift of the ^13^C carboxylic signal observed in the spectra of these adducts confirms the deprotonation of the carboxylic groups and their ionic nature (Table 4). The small difference in ppm observed between the carboxylic signal of **FLU** and **FLU‐TYA** (Δδ(C═O) = 0.4 ppm) is ascribable to the cyclic dimerization of COOH in the pure API, with the formation of a $R_{2}^{2}\left(8\right)$ ring motif characterized by a high frequency ^13^C carboxylic signal (δ(C═O) = 183.4 ppm) falling in the COO^−^ region.^[^ ^38^ ^]^ In contrast, **NAP–TYA** exhibits a larger shift (Δδ(C═O) = 6.1 ppm), as pure **NAP** forms chains via neutral hydrogen bonds with a C(4) motif.^[^ ^39^ ^]^ Also for **KET–TYA**, the formation of a new crystalline phase is confirmed, though with slightly lower crystallinity than the previous cases (full width at half maximum ∼ 150–220 Hz). A 1:1 stoichiometry is assumed based on the number of signals, despite the presence of additional signals in the region associated with the aliphatic carbons of **KET**, likely linked to structural disorder of its methyl group. A different stoichiometry, in fact, would have affected also the other ^13^C signals, and the integration of the signals in the ^1^H NMR spectrum (Figure S27 in the Supporting Information), which, instead, confirms the 1:1 stoichiometry. The formation of a molecular salt is assumed due to the shift of the COOH signal towards higher frequencies, despite the small variation observed (Δδ(C═O) = 0.5 ppm) (Table 4). As previously reported for **FLU**, this behavior can be ascribed to the dimerization through carboxylic $R_{2}^{2}\left(8\right)$ homosynthon formation in pure **KET**, which is characterized by a high‐frequency carboxylic signal (δ(C═O) = 184.0 ppm).^[^ ^8^ ^]^

**Table 4:** ^13^C chemical shifts (ppm) of the carboxyl signals of pure APIs and NSAIDs‐TYA adducts.

	δ_iso_ (C═O) ppm		
	Pure API	Adduct	Δδ(C═O)
**NAP**	178.6	184.7	6.1
**FLU**	183.4	183.8	0.4
**KET**	183.5	184.0	0.5
**DIF**	175.1	173.8	1.3

The ^13^C CPMAS analysis of **DIF‐TYA Form II** confirmed the formation of the adduct with a stoichiometry of 1:1, characterized by a good degree of crystallinity (full width at half maximum ∼ 120–190 Hz). The carboxylic ^13^C signal shifts to lower frequency (Δδ(C═O) = 1.3 ppm) compared to pure **DIF** (δ(C═O) = 175.1 ppm) (Table 4). Since $R_{2}^{2}\left(8\right)$ homodimeric interactions between COOH groups are established in pure **DIF**, the small shift to lower frequency suggests a transition from a carboxylate‐like situation, i.e., the cyclic dimerization, to a neutral COOH involved in hydrogen bonds.^[^ ^39^ ^]^ The ^13^C CPMAS analysis therefore points toward the formation of a co‐crystal, albeit strongly discordant with the Δ*pK_a_* rule outcome.

**Figure 5:**
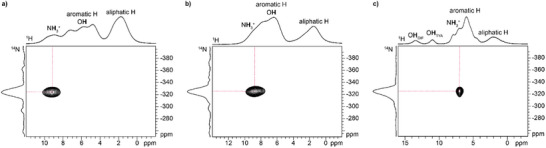
^14^N‐^1^H D‐HMQC (1000.40 MHz) spectra of (a) NAP‐TYA, (b) KET‐TYA, and (c) DIF‐TYA Form II acquired with a spinning speed of 40 kHz at room temperature. The correlations between the nitrogen atom and the protons directly coordinated are represented by red dashed lines.

To further elucidate the nature and the structure of **KET–TYA** and **DIF‐TYA Form II**, ^14^N‐^1^H dipolar HMQC (D‐HMQC), ^1^H MAS, and ^1^H double quantum–single quantum (DQ–SQ) MAS experiments were performed. To simplify the interpretation of the obtained results, the spectra of **NAP–TYA** were also acquired since its structure was fully characterized by SCXRD and ^13^C CPMAS SSNMR analyses. ^14^N‐^1^H D‐HMQC experiments allow to observe protons that are spatially close to nitrogen atoms by exploiting the heteronuclear dipolar interaction between ^1^H and ^14^N.^[^ ^40^ ^]^ The cross‐peaks observed in the spectra of the three supramolecular adducts (Figure 5a,b,c) enabled to assign the amine proton signals at 9.2, 8.8, and 7.0 ppm for **NAP–TYA**, **KET–TYA**, and **DIF‐TYA Form II**, respectively. These chemical shifts are also indicative of the strength of the hydrogen bonds in which they are involved, i.e., the higher the frequency, the stronger the interaction. As a result, in the **KET–TYA** system, the amine group is involved in hydrogen bond interactions of similar strength to that in **NAP–TYA** and stronger than in **DIF‐TYA Form II**.

**Figure 6:**
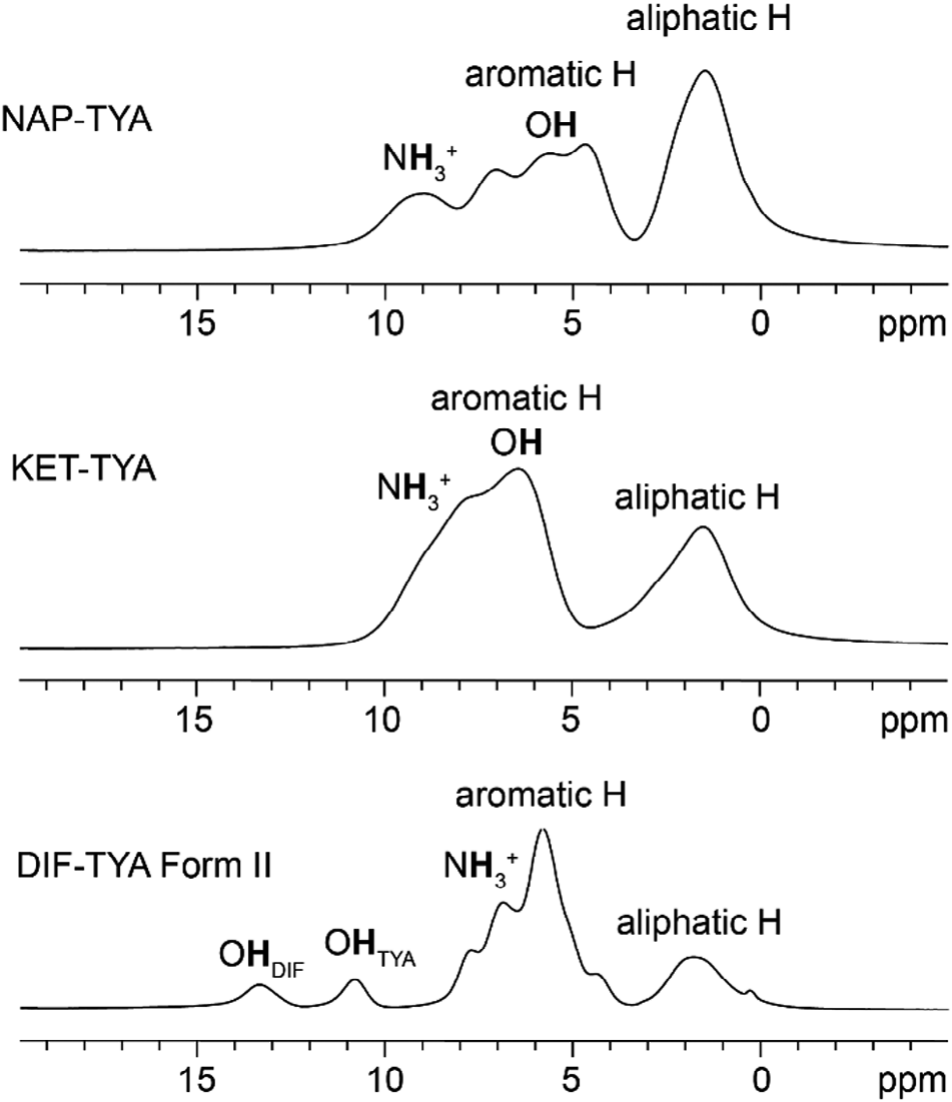
^1^H MAS (1000.40 MHz) spectra of NAP‐TYA (top), KET‐TYA (middle), and DIF‐TYA Form II (bottom) acquired with a spinning speed of 60 kHz at room temperature.

**Figure 7:**
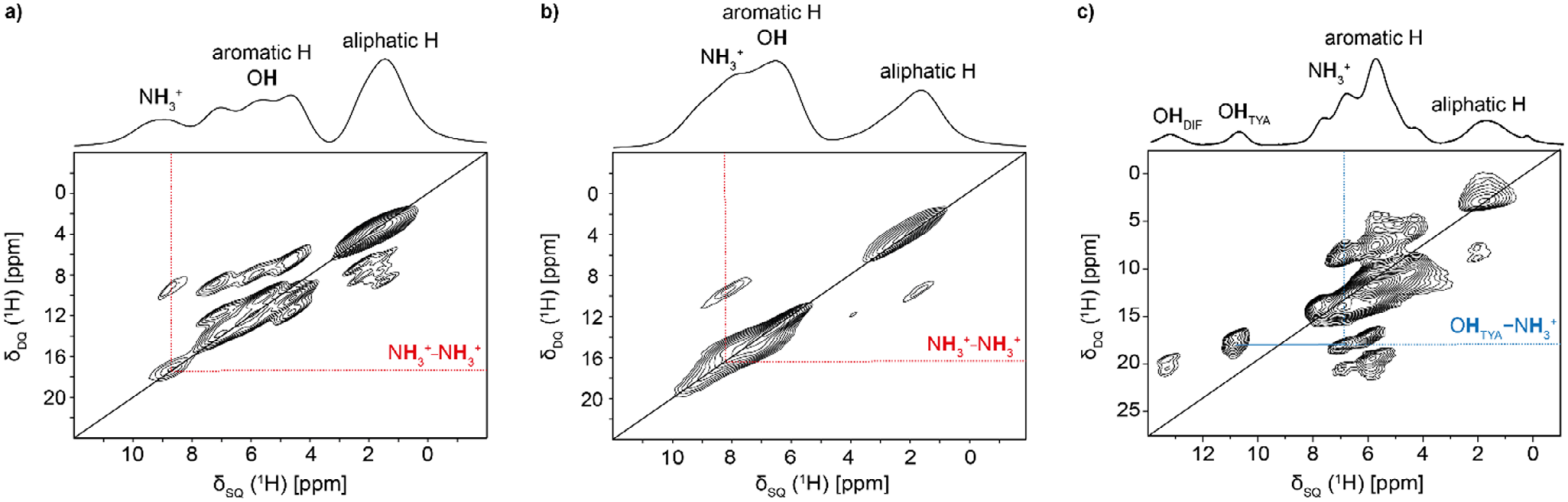
^1^H‐^1^H DQ MAS (1000.40 MHz) spectra of (a) NAP‐TYA, (b) KET‐TYA, and (c) DIF‐TYA Form II acquired with a spinning speed of 60 kHz at room temperature. NH_3_^+^ auto‐peaks are highlighted by red dashed lines. (c) The blue dashed line highlights the proximity between the hydroxylic and the aminic protons of two different molecules of TYA.

The protonation of the amine group in **KET–TYA** and **DIF‐TYA Form II** was confirmed by integrating the signal associated with the amine protons in their ^1^H MAS spectra (Figure 6), thereby establishing the ionic nature of the two adducts. Moreover, in the ^1^H MAS spectrum of **DIF‐TYA Form II** two signals at higher frequencies are observed. The one at 10.8 ppm belongs to the proton of the hydroxyl group of **TYA** involved in the hydrogen bond interaction with the carboxyl group of **DIF** (O∙∙∙O = 2.652(2) Å). The peak at 13.3 ppm is attributed to the proton of the hydroxyl group of **DIF**, whose high chemical shift is indicative of its involvement in the strong S(6) intramolecular hydrogen bond interaction (O∙∙∙O = 2.555(7) Å), already present in the pure API. The ^1^H DQ‐SQ MAS spectra (Figure 7) were used to evaluate the proton‐proton proximities to obtain further structural information for completing the crystallographic data, especially for **KET–TYA**. The presence of a ${\mathrm{NH}}_{3}^{+}$ auto‐peak in the ^1^H DQ–SQ MAS spectra of both **NAP–TYA** (Figure 7a; δ_DQ_ = 18 ppm; N**H**_3_^+^_TYA_∙∙∙ N**H**_3_^+^_TYA_ = 2.865–3.396 Å) and **KET–TYA** (Figure 7b; δ_DQ_ = 17 ppm) suggests the formation of a $R_{4}^{4}\left(10\right)$ ring motif connecting 4 molecules also in **KET–TYA**. For **DIF‐TYA Form II**, the observed proximities confirm the crystallographic structure. Emphasis is placed on the proximity between the protons of the hydroxyl and the amine group of **TYA** (Figure 7c; δ_DQ_ = 18 ppm; N**H**_3_^+^_TYA_∙∙∙O**H**_TYA_ = 2.356(8) Å)), which confirms the establishment of the 2D hydrogen bonds network.

### 2.5 Solubility Tests

While the most widely used method to quantify the solubility of pharmaceutical compounds is HPLC‐UV, solution ^1^H NMR experiments have emerged as a powerful alternative for quantitative analysis due to several advantages: (i) quantification can be performed using an internal standard without requiring calibration curves, (ii) although the low sensitivity of NMR could be seen as a problem in limiting analysis of samples with low solute concentrations, the aqueous solubility of pharmaceutical compounds typically exceeds the detection limit.^[^ ^23^, ^41^, ^42^, ^43^, ^44^, ^45^, ^46^ ^]^ In this study, ^1^H NMR experiments were performed to compare the aqueous solubility of the drugs in the pure form and in the adducts. Isolated and nonoverlapping signals for the pure NSAIDs and the multicomponent crystalline forms were selected (Supporting Information, Tables S7 and S8) and integrated in relation to the signal of the internal standard TSP (1 mg/mL), to calculate the concentration (mg/mL) of the drug in solution for each system. The results obtained in aqueous solution highlight an effective improvement in aqueous solubility for all the adducts; specifically, a 35‐fold increase is observed for **NAP–TYA**, 44‐fold for **FLU–TYA**, 22‐fold for **KET–TYA**, and 17‐fold for **DIF‐TYA Form II** (Supporting Information, Table S9).

## 3 Conclusions

The use of knowledge‐based methods for the synthesis of supramolecular adducts led to the discovery of an attractive co‐former, **TYA**, for the formation of new multicomponent systems with NSAIDs. The achievement of three new molecular salts, **NAP–TYA**, **FLU–TYA**, and **KET–TYA** demonstrates the robustness of the amino‐carboxylic acid heterosynthon, regardless of the API chemical structure. In the presence of the basic co‐former, the COOH∙∙∙N_aliphatic_ interaction is preferred over the carboxylic homodimers or, in the case of **NAP**, over the chains between neutral COOH. The presence of an additional hydrogen bond functionality on **TYA**, the hydroxyl group, in most cases, enables the establishment of a 2D hydrogen bond network. Moreover, the employment of mechanochemical techniques, which are greener than the solvent‐based ones, results in a new polymorphic form for **DIF‐TYA**, i.e., Form II. Obtaining this new phase, despite one already reported, highlights once again the importance of searching for new crystal forms using the widest possible range of different synthetic techniques and conditions.

The adducts obtained were extensively characterized using various techniques, among which SCXRD and SSNMR played the major role. The combination of these methods afforded an in‐depth structural characterization and the analysis of the ionic nature of the adducts. The use of ^1^H MAS and SSNMR experiments enabled by fast spinning, such as the ^14^N‐^1^H D‐HMQC, and ^1^H DQ‐SQ MAS, was decisive to deeper investigate the **KET–TYA** structure, for which SCXRD analysis could not be performed. For **DIF‐TYA Form II**, SSNMR analyses definitively confirmed its ionic nature, which was not clear from SCXRD or ^13^C CPMAS alone. Aqueous solubility tests, carried out by using solution ^1^H NMR experiments, showed a remarkable increase in the equilibrium solubility for all the adducts analyzed. These promising results show how multicomponent crystalline forms affect and enhance the physicochemical properties, in this case solubility, of APIs.

In conclusion, this study contributes to the development of a standardized procedure for producing new multicomponent crystalline systems, from design to characterization, with applications across various fields. Solid‐state and solution NMR have proven to be powerful techniques in this field, demonstrating their dual role in structural characterization and the investigation of physicochemical properties.

## 4 Experimental Section

### 4.1 Materials

**NAP** (*S*‐Naproxen, (S)‐(+)‐2‐(6‐methoxy‐2‐naphthyl)propionic acid, 99%) was purchased from Alfa Aesar (US), while **KET** (Ketoprofen, (±)‐2‐(3‐benzoylphenyl)propionic acid, > 98%), **FLU** (Flurbiprofen, (±)‐2‐(2‐fluorobiphenyl‐4‐yl)propionic acid, > 98%), and **TYA** (Tyramine, 4‐(2‐aminoethyl)phenol, > 98%) from Tokyo Chemical Industry (TCI, Milan, Italy). **DIF** (Diflunisal, 2′,4′‐difluoro‐4‐hydroxy‐[1,1′‐biphenyl]‐3‐carboxylic acid) was purchased from Sigma Aldrich. All reagents were used as such without further purification.

### 4.2 Rational Design

**Full Interaction Maps (FIMs)**. Full Interaction Maps of **IBU**, **NAP**, **FLU**, **KET**, and **DIF** were generated from the crystal structures IBPRAC, COYRUD13, FLUBIP, KEMRUP, and FAFWIS02, respectively, using the full interaction maps tool in CSD‐Materials (Mercury 2024.1.0). The crystal structures selected are the one of the experimental solid forms used (Supporting Information, Figures S7–S12). The analysis considered amine groups, hydroxyl groups, and π–π stacking interactions.

### 4.3 Motif Search

The motif search functionality in CSD‐materials (Mercury 2024.1.0) was used to evaluate the likelihood of intermolecular hydrogen bond interactions involving COOH···OH phenolic and COOH···NH_2_ aliphatic contacts. The search criteria included intermolecular hydrogen bonds with heavy‐atoms distances shorter than 3.5 Å and bond angles greater than 120°. All possible intermolecular hydrogen bonds motifs were considered.

### 4.4 Molecular Complementarity (MC)

MC analysis was performed for **NAP–TYA**, **FLU–TYA**, and **KET–TYA** using the screen by molecular complementarity tool in CSD‐Materials (Mercury 2024.1.0). The tool directly reports the percentage probability of the co‐crystal formation: the higher the percentage value, the greater the probability of positive experimental results. The percentage is given by the number of different API conformations that are complementary with the co‐former, assessing shape, size, and interactions sites of the two molecules. Ten different conformations of **NAP**, **FLU**, and **KET** were evaluated based on the crystal structures COYRUD13, FLUBIP, and KEMRUP, respectively. For **TYA** the crystal structure SENJEC was used.

### 4.5 Preparation Techniques

**NAP–TYA**, **FLU–TYA**, and **KET–TYA** were obtained by kneading. A 1:1 ratio of APIs and **TYA** (Supporting Information, Table S10) was milled in a mortar and pestle for 15 min adding three aliquots of 1 mL of ethanol as solvent for **NAP–TYA** and **FLU–TYA**, and methanol for **KET–TYA**. Single crystals suitable for SCXRD were obtained for **NAP–TYA** and **FLU–TYA** by dissolving 10 mg of each API and one equivalent of **TYA** in the minimum amount of methanol and ethanol, respectively, and then by adding a nucleation seed of the corresponding preformed adduct. The crystallization was achieved, after 7 days, by evaporation of the solvent at room temperature. **DIF‐TYA Form II** was synthesized by grinding **DIF** and **TYA** in a 1:1 ratio (Supporting Information, Table S5) in a mortar and pestle for 30 min. Single crystals suitable for SCXRD were obtained after 7 days of slow diffusion of cyclohexane vapors in an ethanol solution of the adduct (10 mg) at room temperature.

### 4.6 IR Spectroscopy

Fourier transform infrared (FT‐IR) spectra were recorded on an Equinox 55 spectrometer with an ATR reflectance attachment. Spectra were collected in the 400–4000 cm^−1^ range with a resolution of 4 cm^−1^ and 16 scans.

### 4.7 Powder X‐ray Diffraction

X‐ray powder patterns of the powdered samples were acquired on a Philips X'pert PW3020 diffractometer in the Bragg–Brentano geometry, using Cu‐Kα X‐radiation (*λ* = 1.54506 Å) at 40.0 kV and 30.0 mA power. The measurements were collected in θ/2θ mode over the 2*θ* range 5°–50°.

### 4.8 Single‐Crystal X‐ray Diffraction

Selected single crystal samples of **NAP–TYA**, **FLU–TYA**, and **DIF‐TYA Form II** were mounted on a glass fiber and diffraction data were collected at room temperature on a Xcalibur, AtlasS2, Gemini Ultra diffractometer using graphite monochromated Cu‐Kα radiation (*λ* = 1.54506 Å). The CrysAlisPro (Rigaku OD, 2015) software was used for retrieving cell parameters, for performing data reduction, and for absorption correction (with multiscan technique). All structures were solved by direct methods using ShelXS‐14^[^ ^47^ ^]^ and refined with full‐matrix least‐squares on F^2^ using SHELXL‐14^[^ ^48^ ^]^ and the Olex2 program.^[^ ^49^ ^]^ All nonhydrogen atoms were anisotropically refined. Hydrogen atoms were calculated and riding on the corresponding atom. Structure images were obtained using the Mercury software.^[^ ^50^ ^]^

### 4.9 Solid‐State NMR

The ^13^C CPMAS spectra of **DIF**, **TYA**, **NAP–TYA**, **FLU–TYA**, and **KET‐TYA** were acquired with a Bruker Avance II 400 Ultra Shield instrument, operating at 400.23 and 100.63 MHz for ^1^H and ^13^C nuclei, respectively. The powder samples were packed directly into 4 mm zirconia rotors without further preparation. The spectra were acquired at room temperature at a spinning rate of 12 kHz, using a ramped cross‐polarization pulse sequence with a 90° ^1^H pulse of 3.90 µs, and a contact time of 3 ms. The optimized recycle delays (T_1_^1^H*1.27) were between 1.27 and 400 s and the number of scans was in the range of 140–930 depending on the sample. The two‐pulse phase modulation (TPPM) decoupling scheme was used, with a radiofrequency field of 65.7 kHz. The ^13^C chemical shift scale was calibrated through the signals of adamantane (δ(^13^C) 38.48 ppm with reference to methylenic peak). For **NAP**, **FLU**, **KET**, and **DIF‐TYA Form II**, the ^13^C CPMAS spectra were collected with a Jeol ECZR 600 instrument, operating at 600.17 and 150.91 MHz for ^1^H and ^13^C nuclei, respectively. The powder samples were packed directly into 3.2 mm zirconia rotors without further preparation. The spectra were acquired at room temperature at a spinning speed of 20 kHz, using a ramped cross‐polarization pulse sequence with a 90° ^1^H pulse of 2.2 µs, a contact time of 3.5 ms, optimized recycle delays (T_1_^1^H*1.27) between 2 and 6 s, and a number of scans in the range of 50–1450 depending on the sample. The TPPM decoupling scheme was used, with a radiofrequency field of 108.5 kHz. The ^13^C chemical shift scale was calibrated through the signals of adamantane (δ (^13^C) 38.48 ppm with reference to methylenic peak).

The 1D ^1^H, 2D ^1^H DQ‐SQ MAS, and ^14^N‐^1^H D‐HMQC spectra of **NAP–TYA**, **KET–TYA**, and **DIF‐TYA Form II** were acquired with a Bruker Ascend (23.4 T) instrument, operating at 1000.40 MHz. The powder samples, without further preparation, were packed into 1.3 mm zirconia rotors for ^1^H MAS and ^1^H DQ‐SQ MAS, and into 1.9 mm zirconia rotors for ^14^N‐^1^H D‐HMQC. ^1^H MAS and ^1^H DQ MAS spectra were acquired at room temperature at a spinning speed of 60 kHz. The 2D ^1^H DQ‐SQ MAS experiments were performed with the back‐to‐back (BABA) recoupling pulse sequence with excitation time durations of one rotor period (^1^H 90° = 2.5 µs). The ^1^H chemical shift scale was indirectly calibrated from the calibration of the ^13^C scale via the ^13^C' signal of alanine (δ (^13^C) 177.8 ppm). For the ^14^N‐^1^H D‐HMQC spectra, instead, heteronuclear dipolar couplings were reintroduced by applying rotary resonance recoupling (*R*_3_) under the *n* = 2 condition, as proposed by Gan et al.^[^ ^51^ ^]^ using *x*−*x* phase inversion. Each recoupling block had a length of 25 µs. τ_mix_ of 200 µs were used. The PM pulse length was 0.6 ms and the ^14^N field was 40 kHz (calibrated with NH_4_Cl).

### 4.10 Solubility Tests

15 mg of each APIs and the corresponding amount of adducts (Supporting Information, Table S11) were introduced into vials with 4 mL of deionized H_2_O. The samples were stirred for 3 days, centrifuged at 4500 rpm for 2 min to separate excess precipitate, and filtered using 0.20 µm Millipore PTFE syringe filters. For the quantification, a solution of TSP (3‐(trimethylsilyl)‐propionic acid 2,2,3,3‐d_4_ sodium salt) in deuterated water (1 mg/mL in D_2_O) was used as internal standard. The TSP was purchased from Alfa Aesar, and the deuterated water (D_2_O, 99.96% D, for NMR spectroscopy) from VWR Chemicals. 540 µL of the filtered samples and 60 µL of the internal standard were introduced in the NMR tube. Quantitative analysis was performed by selecting isolated, nonoverlapping peaks in both pure APIs and adducts, reported in Tables S7 and S8 in the Supporting Information, and integrating them in relation to the internal standard TSP (*δ* = 0 ppm, 9H) at a known concentration (1 mg/mL, integral = 0.9). The concentration (mg/mL) of each sample in water was then determined by calculating the ratio between the signal integrations of the APIs in pure form or in the adducts and the number of nuclei responsible for the specific resonances. Solution ^1^H NMR spectra were acquired on a Jeol ECZR 600 instrument operating at 600.17 MHz for ^1^H. The spectra were acquired at 298 K, using 64 scans, an acquisition time of 2.90 s, a resolution of 0.4 Hz, and a relaxation delay of 60 s. The water signal was suppressed using the pre‐saturation scheme with an offset of 4.665 ppm and an attenuation of 60 db. For each sample, three measures were performed.

## Supporting Information

Additional references cited within the Supporting Information.^[^ ^52^, ^53^, ^54^, ^55^ ^]^ The crystallographic data for the crystallized compounds were deposited within the Cambridge Crystallographic Data Centre as supplementary publications under the CCDC numbers 2383061 (NAP‐TYA), 238302 (FLU‐TYA), and 2383063 (DIF‐TYA Form II). These data are provided free of charge by the Cambridge Crystallographic Data Centre Access Structures service via https://www.ccdc.cam.ac.uk/data_request/cif.

## Conflict of Interest Statement

The authors declare no conflict of interest.

## Supporting information



Supporting Information

## Data Availability

The data that support the findings of this study are available in the Supporting Information of this article.

## References

[1] U. Garg , Y. Azim , RSC Med. Chem. 2021, 12, 705–721. 34124670 10.1039/d0md00400f PMC8152597

[2] A. L. C. S. Nascimento , R. P. Fernandes , M. D. Charpentier , J. H. ter Horst , F. J. Caires , M. Chorilli , Particuology 2021, 58, 227–241.

[3] G. Bolla , B. Sarma , A. K. Nangia , Chem. Rev. 2022, 122, 11514. 35642550 10.1021/acs.chemrev.1c00987

[4] N. Shan , M. J. Zaworotko , Drug Discov. Today 2008, 13, 440. 18468562 10.1016/j.drudis.2008.03.004

[5] M. Guo , X. Sun , J. Chen , T. Cai , Acta Pharm. Sin. B 2021, 11, 2537. 34522597 10.1016/j.apsb.2021.03.030 PMC8424375

[6] V. K. Nanjwade , F. V. Manvi , M. Shamrez Ali , B. K. Nanjwade , M. M. Maste , J. Appl. Pharm. Sci. 2011, 1, 1.

[7] G. Varrassi , E. Alon , M. Bagnasco , L. Lanata , V. Mayoral‐Rojals , A. Paladini , J. V. Pergolizzi , S. Perrot , C. Scarpignato , T. Tölle , Adv. Ther. 2019, 36, 2618. 31485978 10.1007/s12325-019-01053-x PMC6822819

[8] A. Aramini , G. Bianchini , S. Lillini , S. Bordignon , M. Tomassetti , R. Novelli , S. Mattioli , L. Lvova , R. Paolesse , M. R. Chierotti , M. Allegretti , Pharm. Basel Switz. 2021, 14, 555. 10.3390/ph14060555 PMC8230491 34200917

[9] W. Martin , G. Koselowske , H. Töberich , T.h. Kerkmann , B. Mangold , J. Augustin , Biopharm. Drug Dispos. 1990, 11, 265. 2109643 10.1002/bdd.2510110311

[10] I. Nugrahani , D. Utami , S. Ibrahim , Y. P. Nugraha , H. Uekusa , Eur. J. Pharm. Sci. 2018, 117, 168. 29475066 10.1016/j.ejps.2018.02.020

[11] G. R. Desiraju , Angew. Chem. Int. Ed. Engl. 1995, 34, 2311.

[12] G. R. Desiraju , J. Chem. Sci. 2010, 122, 667.

[13] S. Aitipamula , R. Banerjee , A. K. Bansal , K. Biradha , M. L. Cheney , A. R. Choudhury , G. R. Desiraju , A. G. Dikundwar , R. Dubey , N. Duggirala , P. P. Ghogale , S. Ghosh , P. K. Goswami , N. R. Goud , R. R. K. R. Jetti , P. Karpinski , P. Kaushik , D. Kumar , V. Kumar , B. Moulton , A. Mukherjee , G. Mukherjee , A. S. Myerson , V. Puri , A. Ramanan , T. Rajamannar , C. M. Reddy , N. Rodriguez‐Hornedo , R. D. Rogers , M. J. Zaworotko , Cryst. Growth Des. 2012, 12, 2147.

[14] A. J. Cruz‐Cabeza , CrystEngComm 2012, 14, 6362.

[15] S. Tothadi , T. R. Shaikh , S. Gupta , R. Dandela , C. P. Vinod , A. K. Nangia , Cryst. Growth Des. 2021, 21, 735.

[16] S. N. Wong , Y. C. S. Chen , B. Xuan , C. C. Sun , S. F. Chow , CrystEngComm 2021, 23, 7005.

[17] P. A. Wood , N. Feeder , M. Furlow , P. T. A. Galek , C. R. Groom , E. Pidcock , CrystEngComm 2014, 16, 5839.

[18] L. Fábián , Cryst. Growth Des. 2009, 9, 1436.

[19] S. Mittapalli , M. K. C. Mannava , R. Sahoo , A. Nangia , Cryst. Growth Des. 2019, 19, 219.

[20] A. Gunnam , A. K. Nangia , CrystEngComm 2021, 23, 3398.

[21] D. E. Boycov , A. N. Manin , K. V. Drozd , A. V. Churakov , G. L. Perlovich , CrystEngComm 2022, 24, 2280.

[22] M. Li , W. Xu , Y. Su , TrAC Trends Anal. Chem. 2021, 135, 116152.

[23] M. Zloh , ADMET DMPK 2019, 7, 242. 35359618 10.5599/admet.737 PMC8963582

[24] C. B. Aakeröy , Acta Crystallogr. B 1997, 53, 569.

[25] P. Vishweshwar , J. A. McMahon , M. L. Peterson , M. B. Hickey , T. R. Shattock , M. J. Zaworotko , Chem. Commun. Camb. Engl. 2005, 36, 4601. 10.1039/b501304f 16158128

[26] R. Parveen , P. Dastidar , Chem.–Eur. J. 2016, 22, 9257. 27226393 10.1002/chem.201600105

[27] R. Parveen , P. Dastidar , Chem.–Asian J. 2015, 10, 2427. 26247886 10.1002/asia.201500732

[28] M. Rodrigues , B. Baptista , J. A. Lopes , M. C. Sarraguça , Int. J. Pharm. 2018, 547, 404. 29890258 10.1016/j.ijpharm.2018.06.024

[29] M. Karimi‐Jafari , L. Padrela , G. M. Walker , D. M. Croker , Cryst. Growth Des. 2018, 18, 6370.

[30] E. Pindelska , A. Sokal , W. Kolodziejski , Adv. Drug Deliver. Rev. 2017, 117, 111. 10.1016/j.addr.2017.09.014 28931472

[31] S. Riegelman , L. A. Strait , E. Z. Fischer , J. Pharm. Sci. 1962, 51, 129. 14492164 10.1002/jps.2600510210

[32] P. C. Vioglio , M. R. Chierotti , R. Gobetto , Adv. Drug Deliver. Rev. 2017, 117, 86. 10.1016/j.addr.2017.07.001 28687273

[33] D. Braga , L. Maini , G. de Sanctis , K. Rubini , F. Grepioni , M. R. Chierotti , R. Gobetto , Chem.–Eur. J. 2003, 9, 5538. 14639637 10.1002/chem.200304940

[34] R. G. Griffin , A. Pines , S. Pausak , J. S. Waugh , J. Chem. Phys. 1975, 63, 1267.

[35] Z. Gu , A. McDermott , J. Am. Chem. Soc. 1993, 115, 4282.

[36] R. K. Harris , Solid State Sci. 2004, 6, 1025.

[37] M. Geppi , S. Borsacchi , E. Carignani in Disordered Pharmaceutical Materials (Ed.: M. Descamps ), Wiley‐VCH, Germany 2016, pp. 427–466.

[38] G. Mollica , M. Geppi , R. Pignatello , C. A. Veracini , Pharm. Res. 2006, 23, 2129. 16952004 10.1007/s11095-006-9044-z

[39] S. Ando , J. Kikuchi , Y. Fujimura , Y. Ida , K. Higashi , K. Moribe , K. Yamamoto , J. Pharm. Sci. 2012, 101, 3214. 22517167 10.1002/jps.23158

[40] Y. Nishiyama , Y. Endo , T. Nemoto , H. Utsumi , K. Yamauchi , K. Hioka , T. Asakura , J. Magn. Reson. 2011, 208, 44. 21035366 10.1016/j.jmr.2010.10.001

[41] A. Avdeef , C. M. Berger , C. Brownell , Pharm. Res. 2000, 17, 85. 10714613 10.1023/a:1007526826979

[42] M. Lin , M. Tesconi , M. Tischler , Int. J. Pharm. 2009, 369, 47. 19073245 10.1016/j.ijpharm.2008.10.038

[43] S. K. Bharti , R. Roy , TrAC Trends Anal. Chem. 2012, 35, 5.

[44] G. Dikmen , J. Mol. Liq. 2018, 272, 851.

[45] I. Ghiviriga , Anal. Chem. 2023, 95, 2706. 36705621 10.1021/acs.analchem.2c03277

[46] D. Komisarek , E. Taskiran , V. Vasylyeva , Materials 2023, 16, 2242. 36984121 10.3390/ma16062242 PMC10054091

[47] G. M. Sheldrick , Acta Crystallogr. A 2008, 64, 112. 18156677 10.1107/S0108767307043930

[48] G. M. Sheldrick , Acta Crystallogr. Sect. Found. Adv. 2015, 71, 3. 10.1107/S2053273314026370 PMC4283466 25537383

[49] O. V. Dolomanov , L. J. Bourhis , R. J. Gildea , J. A. K. Howard , H. Puschmann , J. Appl. Crystallogr. 2009, 42, 339. 10.1107/S0021889811041161 PMC3236671 22199401

[50] C. F. Macrae , I. J. Bruno , J. A. Chisholm , P. R. Edgington , P. McCabe , E. Pidcock , L. Rodriguez‐Monge , R. Taylor , J. van de Streek , P. A. Wood , J. Appl. Crystallogr. 2008, 41, 466.

[51] Z. Gan , J. P. Amoureux , J. Trébosc , Chem. Phys. Lett. 2007, 435, 163.

[52] E. Carignani , S. Borsacchi , J. P. Bradley , S. P. Brown , M. Geppi , J. Phys. Chem. C 2013, 117, 17731.

[53] J. R. Yates , S. E. Dobbins , C. J. Pickard , F. Mauri , P. Y. Ghi , R. K. Harris , Phys. Chem. 2005, 7, 1402. 10.1039/b500674k 19787961

[54] T. N. Pham , S. A. Watson , A. J. Edwards , M. Chavda , J. S. Clawson , M. Strohmeier , F. G. Vogt , Mol. Pharmaceutics 2010, 7, 1667. 10.1021/mp100205g 20681586

[55] F. G. Vogt , H. Yin , R. G. Forcino , L. Wu , Mol. Pharmaceutics 2013, 10, 3433. 10.1021/mp400275w 23822557

