# Supplementary material for: Exploring Tyramine's Role in the Formation of Supramolecular Adducts with Nonsteroidal Anti‐Inflammatory Drugs
Source: Chemistry. 2025 Apr 3;31(26):e202500080. doi: 10.1002/chem.202500080 (PMC12063043; doi:10.1002/chem.202500080)
Supplement: Supplementary file 1 — Supporting Information [file CHEM-31-e202500080-s001.pdf]

Chemistry – A European Journal
Supporting Information

Exploring Tyramine's Role in the Formation of Supramolecular Adducts with Nonsteroidal Anti-Inflammatory Drugs

Chiara Rosso,[a] Rebecca Birolo,[a] Angelo Gallo,[a] William T. Franks,[b] Emanuele Priola,[a] Michele R. Chierotti,[a] and Roberto Gobetto*[a]

[a] Department of Chemistry, University of Turin, Via P. Giuria 7, 10125, Turin (Italy)
[b] Department of Physics, University of Warwick, Coventry, CV4 7AL, West Midlands (England)

Table of contents.

1. *Rational design of NSAIDs-TYA supramolecular adducts* 1
   2.1 Motif search analysis 1
2. *Experimental Results* 2
   2.1 FTIR-ATR analysis 2
   2.2 X-ray powder patterns 4
3. *Crystal Structure Analysis* 9
4. *NMR analysis* 14
   4.1 Comparison of the ^{13}C CPMAS spectra of the adducts and the starting materials 14
   4.2 ^{1}H NMR spectrum of KET-TYA. 19
5. *Solubility tests* 20
6. *Experimental Section* 21

1. Rational design of NSAIDs-TYA supramolecular adducts

1.1 Motif search analysis

Motif search analysis enabled the investigation of four hydrogen-bond interactions in the formation of supramolecular synthons, specifically when a -COOH group interacts with either a phenolic hydroxyl or an aliphatic amine group. This analysis revealed a greater propensity for the aliphatic $-NH_2$ group to form supramolecular synthons with the -COOH group rather than with the phenolic -OH group, leading to the formation of structures such as the ones reported in Figure S1.

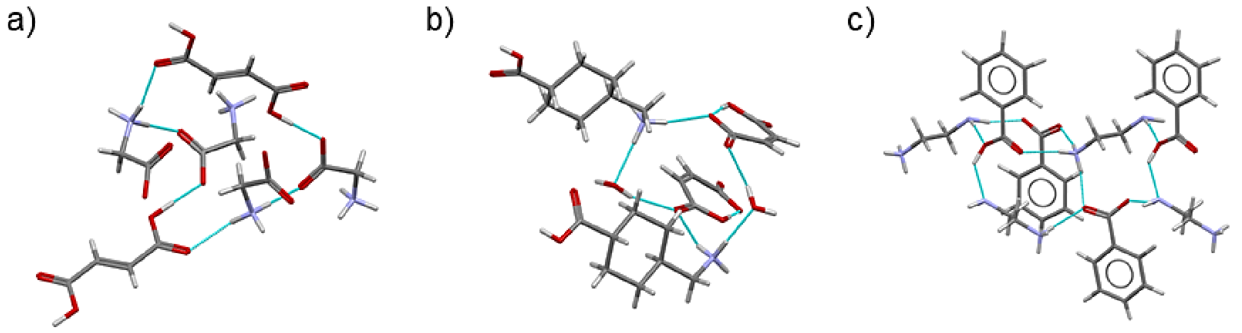

Figure S1. Representative structures from the motif search analysis illustrating interactions between the carboxyl group and the aliphatic amine group. CSD refcodes: a) DOQCOE; b) GIDFUY; c) RIBLET.

However, a good probability (15.1%) of forming interactions between the phenolic hydroxyl group and the C=O moiety of the carboxylic group is also observed. Representative structures illustrating the interaction between the carboxylic group and the phenolic hydroxyl group – considering both the interactions identified as **a** and **b** in the paper – are presented in Figure S2.

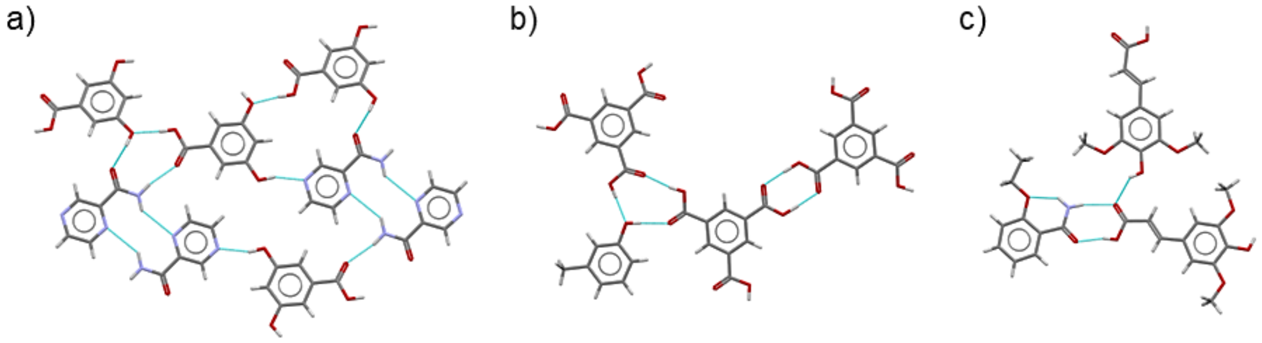

Figure S2. Representative structures from the motif search analysis illustrating interactions between the carboxyl group and the phenolic hydroxyl group. CSD refcodes: a) ACOPOA; b) CAFVUC; c) DEYQUW.

2. Experimental Results

2.1 FTIR-ATR analysis

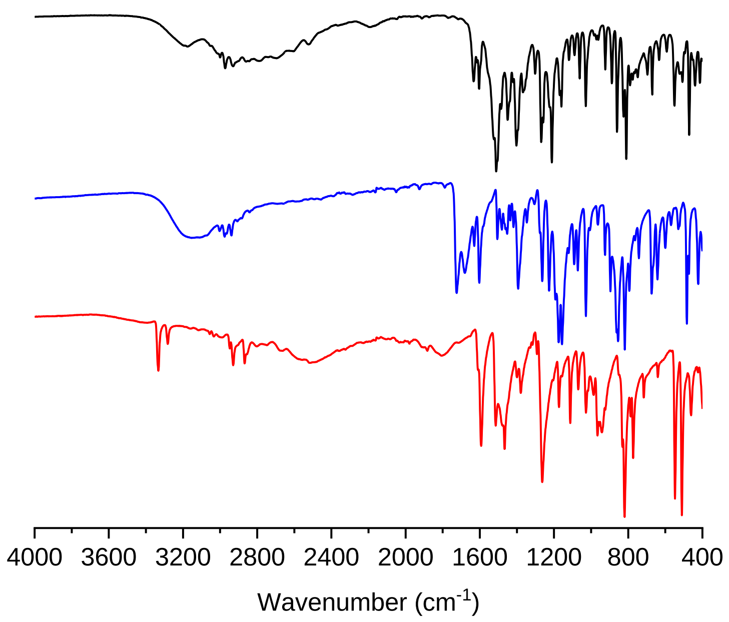

Figure S3. FTIR-ATR spectra of NAP-TYA (black, top), NAP (blue, middle), and TYA (red, bottom).

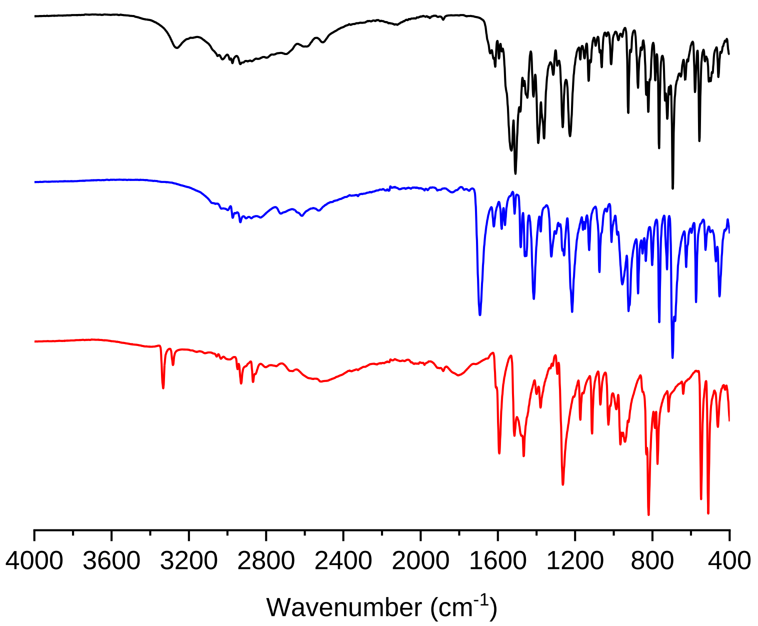

Figure S4. FTIR-ATR spectra of FLU-TYA (black, top), FLU (blue, middle), and TYA (red, bottom).

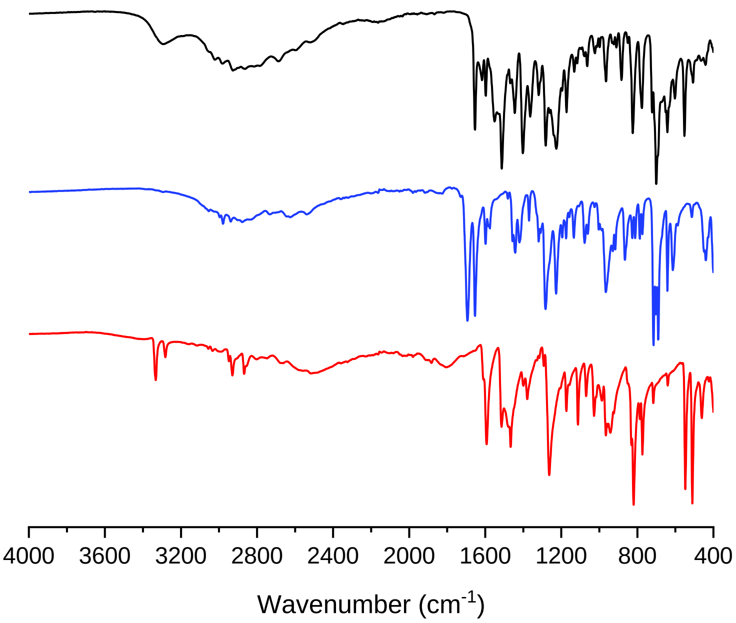

Figure S5. FTIR-ATR spectra of KET-TYA (black, top), KET (blue, middle), and TYA (red, bottom).

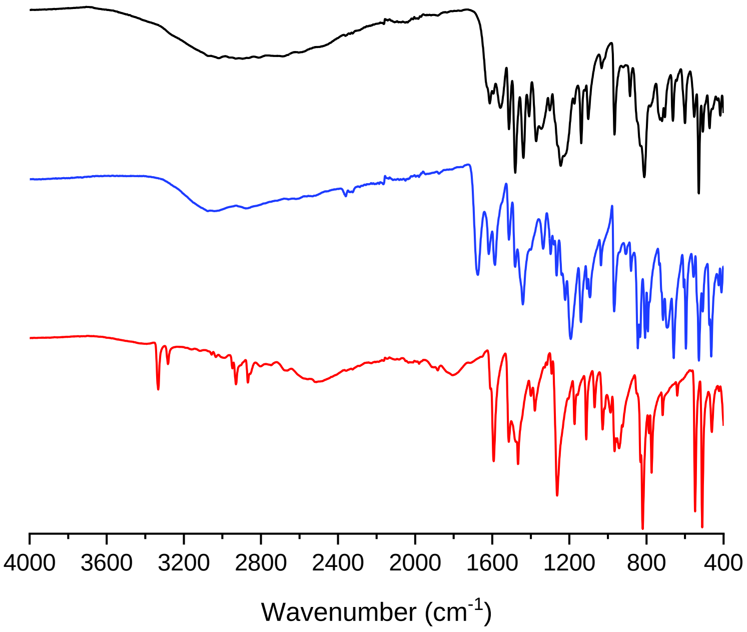

Figure S6. FTIR-ATR spectra of DIF-TYA Form II (black, top), DIF (blue, middle), and TYA (red, bottom).

2.2 X-ray powder patterns

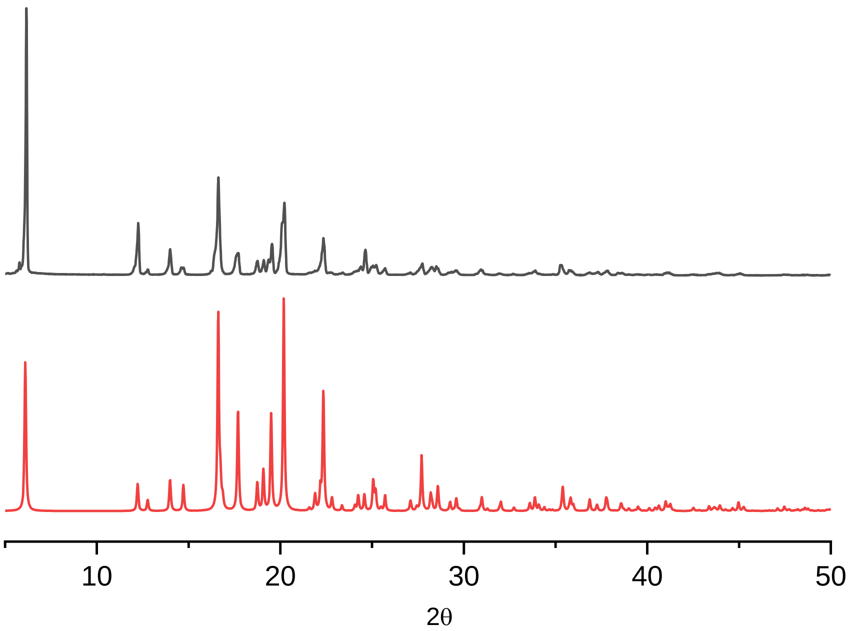

Figure S7. Comparison between the experimental X-ray powder pattern of IBU (black, top) and the one calculated from the crystal structure of IBPRAC (red, bottom).

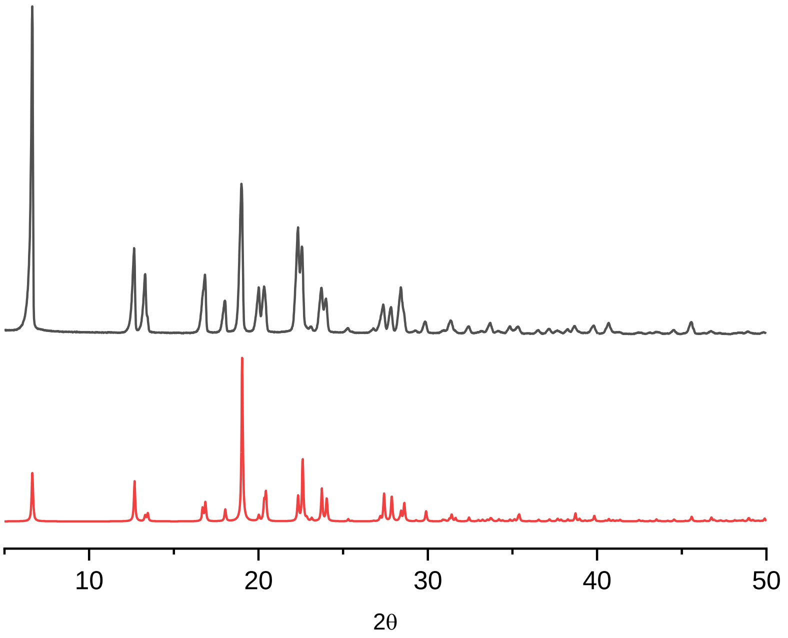

Figure S8. Comparison between the experimental X-ray powder pattern of NAP (black, top) and the one calculated from the crystal structure of COYRUD13 (red, bottom).

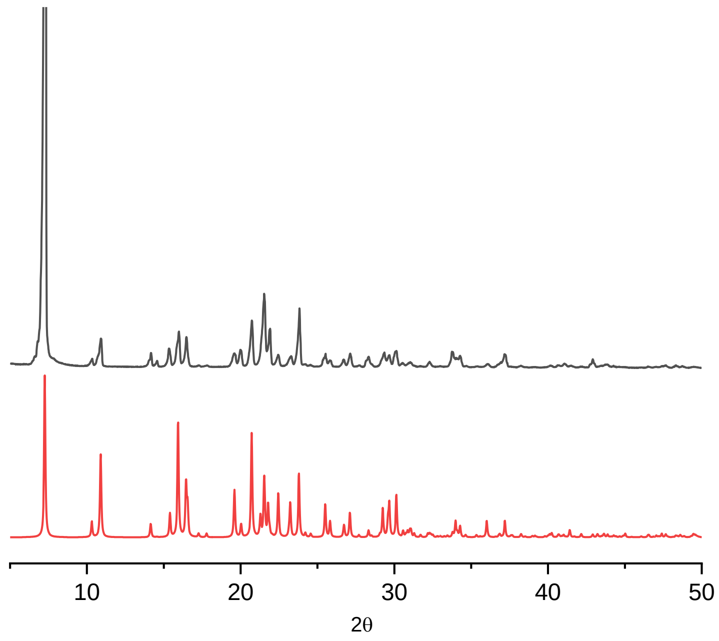

Figure S9. Comparison between the experimental X-ray powder pattern of FLU (black, top) and the one calculated from the crystal structure of FLUBIP (red, bottom).

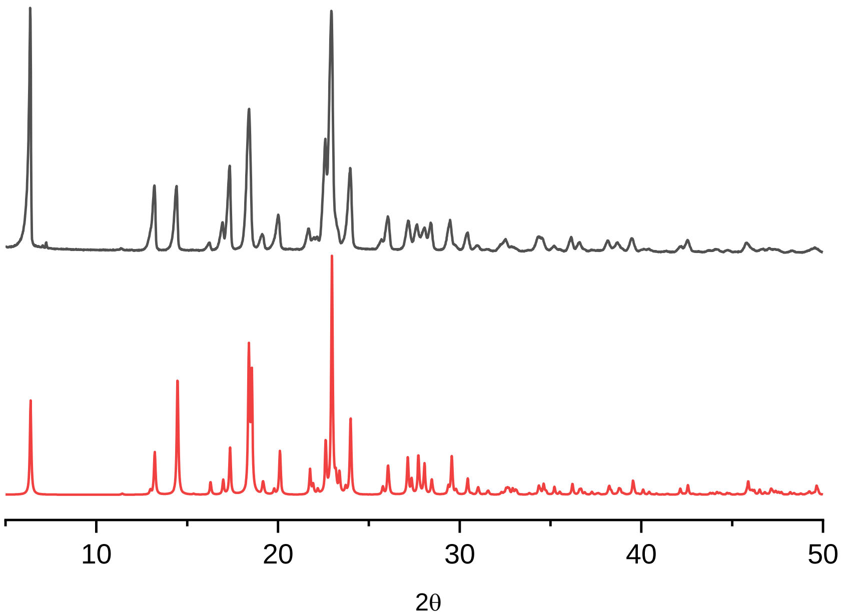

Figure S10. Comparison between the experimental X-ray powder pattern of KET (black, top) and the one calculated from the crystal structure of KEMRUP (red, bottom).

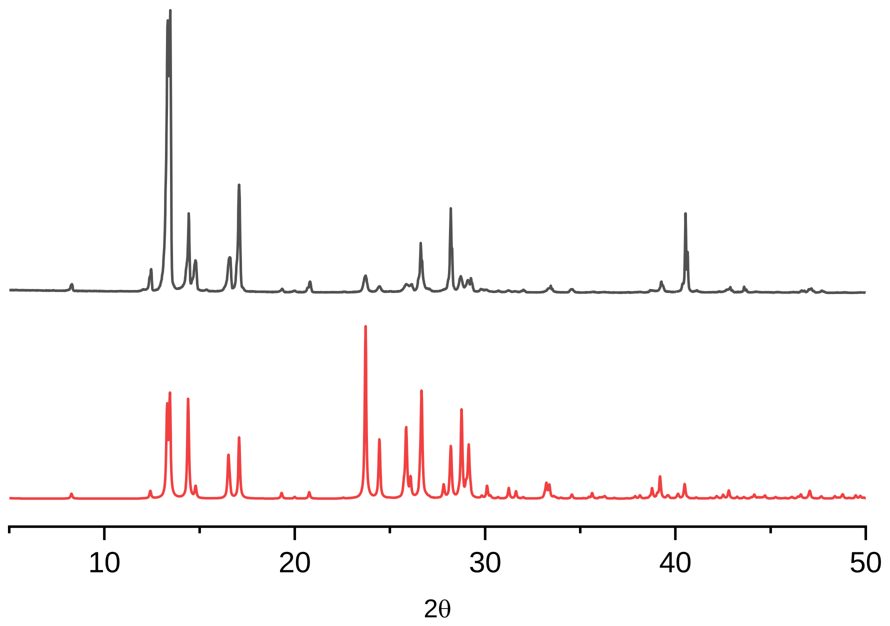

Figure S11. Comparison between the experimental X-ray powder pattern of DIF (black, top) and the one calculated from the crystal structure of FAFWIS01 (red, bottom).

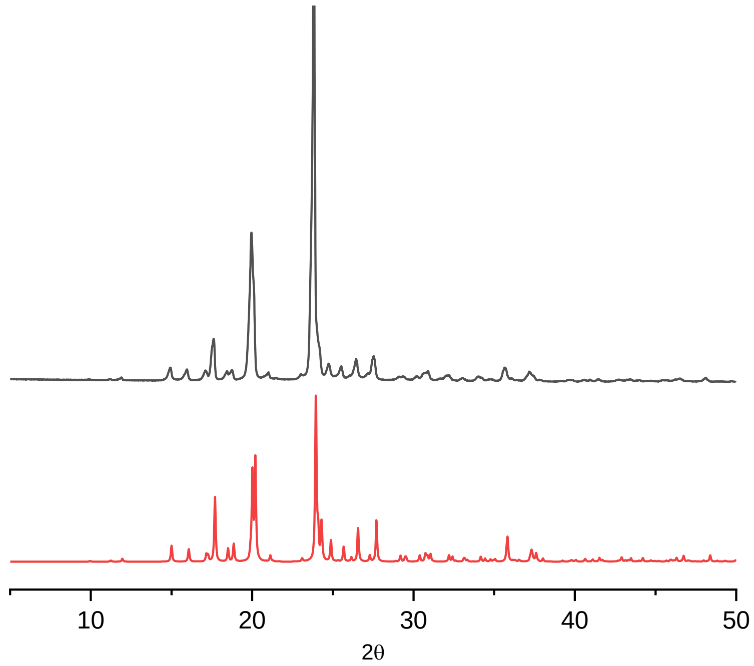

Figure S12. Comparison between the experimental X-ray powder pattern of TYA (black, top) and the one calculated from the crystal structure of SENJEC (red, bottom).

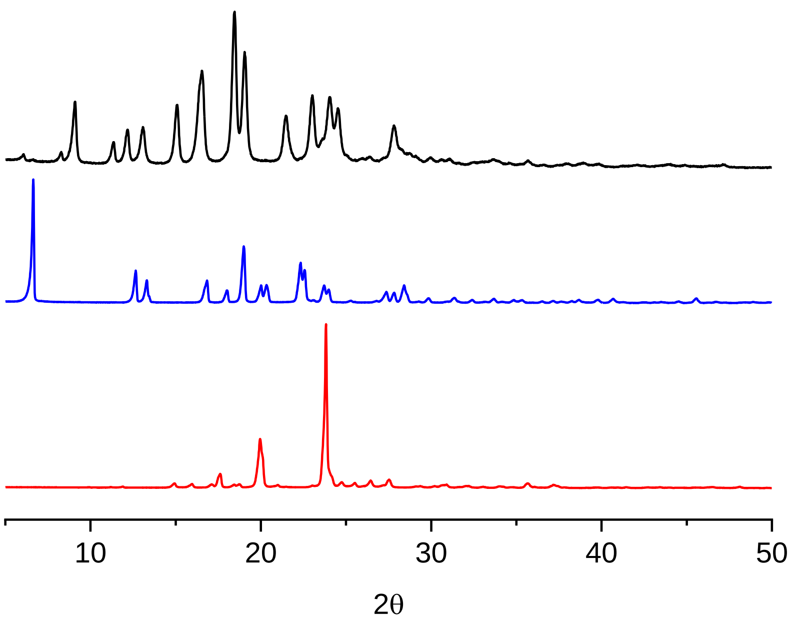

Figure S13. Experimental X-ray powder patterns of NAP-TYA (black, top), NAP (blue, middle), and TYA (red, bottom).

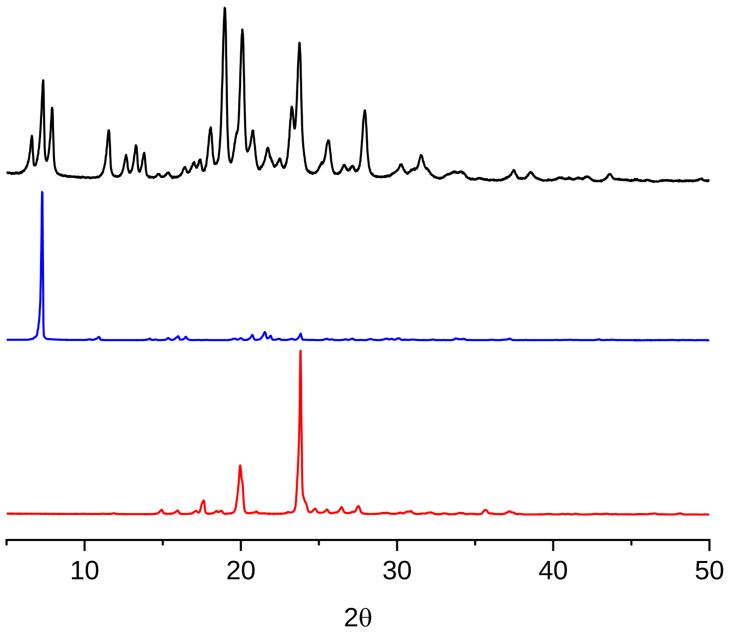

Figure S14. Experimental X-ray powder patterns of FLU-TYA (black, top), FLU (blue, middle), and TYA (red, bottom).

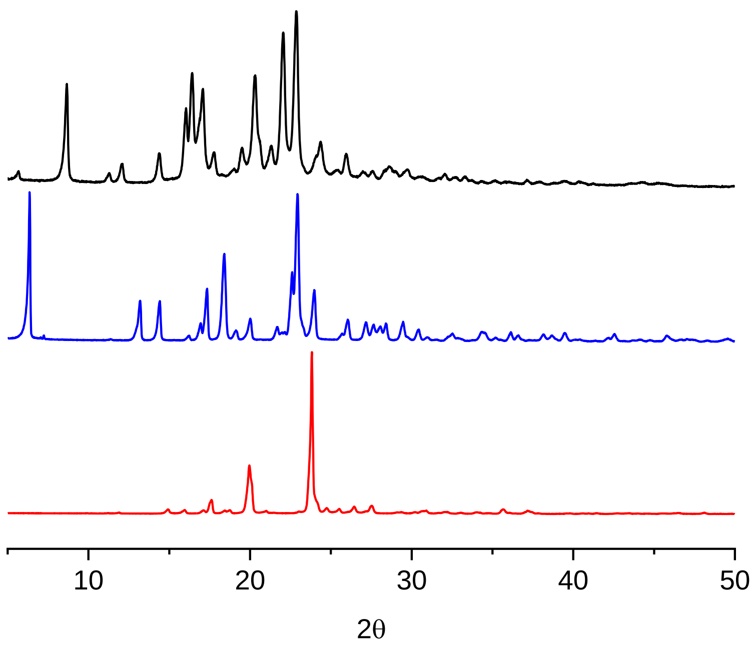

Figure S15. Experimental X-ray powder patterns of KET-TYA (black, top), KET (blue, middle), and TYA (red, bottom).

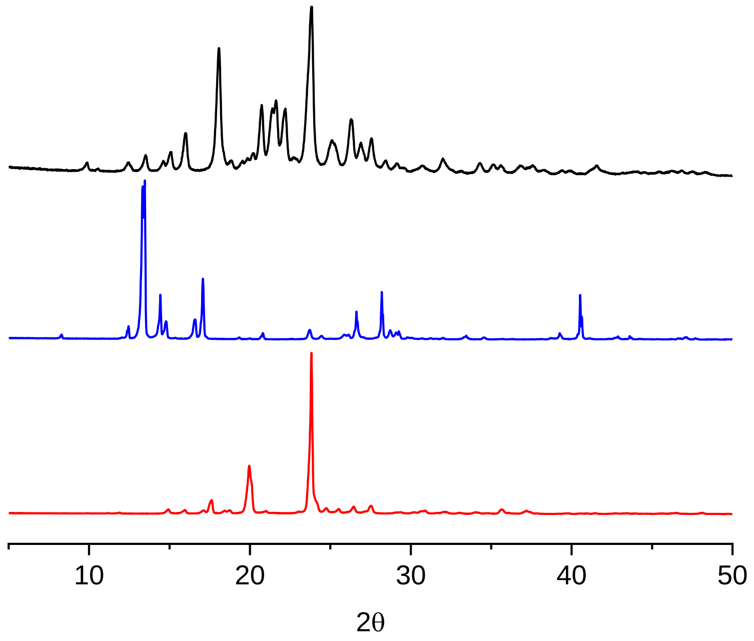

Figure S16. Experimental X-ray powder patterns of DIF-TYA Form II (black, top), DIF (blue, middle), and TYA (red, bottom).

3. Crystal Structure Analysis

Table S1 reports the crystal data collection and the refinement results for the crystal structures of **NAP-TYA**, **FLU-TYA**, and **DIF-TYA Form II**.

Table S1. Crystal data and structure refinement details for NAP-TYA, FLU-TYA, and DIF-TYA Form II.

Identification code	NAP-TYA	FLU-TYA	DIF-TYA Form II
Empirical formula	$C_{22}H_{25}NO_4$	$C_{23}H_{23}FNO_3$	$C_{21}H_{18}F_2NO_4$
Formula weight	367.43	380.42	386.36
Temperature/K	293.00	298.00	298.00
Crystal system	monoclinic	monoclinic	orthorhombic
Space group	$P2_1$	$P2_{1/n}$	*Pbca*
a/Å	10.9076(9)	12.8251(7)	8.7507(3)
b/Å	6.3882(6)	5.8800(3)	17.8363(6)
c/Å	14.8521(13)	26.6340(17)	23.3941(8)
α/°	90	90	90
β/°	103.111(9)	95.641(5)	90
γ/°	90	90	90
Volume/ Å^3	1007.92(16)	1998.8(2)	3651.4(2)
Z	2	4	8
ρ_{calc}g/cm^3	1.211	1.264	1.406
μ/mm^{-1}	0.671	0.734	0.939
F(000)	392.0	804.0	1608.0
Crystal size/mm^3	0.1 × 0.04 × 0.02	0.15 × 0.07 × 0.02	0.22 × 0.11 × 0.08
Radiation	CuKα (λ = 1.54178)	CuKα (λ = 1.54178)	CuKα (λ = 1.54178)
2Θ range for data collection/°	6.11 to 132.93	6.67 to 134.698	7.558 to 133.702
Index ranges	$-12 \leq h \leq 12$<br>$-4 \leq k \leq 7$<br>$-17 \leq l \leq 17$	$-15 \leq h \leq 15$<br>$-6 \leq k \leq 5$<br>$-31 \leq l \leq 31$	$-10 \leq h \leq 10$<br>$-16 \leq k \leq 20$<br>$-26 \leq l \leq 27$
Reflections collected	5942	21186	12382
Indipendent reflections	2605<br>[R_{int} = 0.0351,<br>R_{sigma} = 0.0308]	3489<br>[R_{int} = 0.0722,<br>R_{sigma} = 0.0374]	3189<br>[R_{int} = 0.0337,<br>R_{sigma} = 0.0256]
Data/restraints/parameters	2605/1/249	3489/0/265	3189/0/266
Goodness-of-fit on F^2	1.118	1.061	1.063
Final R indexes [I>=2σ (I)]	R_1 = 0.0433,<br>wR_2 = 0.1134	R_1 = 0.0603,<br>wR_2 = 0.1519	R_1 = 0.0434,<br>wR_2 = 0.1042
Final R indexes [all data]	R_1 = 0.0594,<br>wR_2 = 0.1258	R_1 = 0.0818,<br>wR_2 = 0.1676	R_1 = 0.0589,<br>wR_2 = 0.1161
Largest diff. peak/hole/e Å^{-3}	0.23/-0.12	0.42/-0.22	0.21/-0.18

The single-crystal structures of **NAP-TYA**, **FLU-TYA**, and **DIF-TYA Form II** are representative of the bulk, as it is evident from the comparison of experimental and simulated PXRD patterns (Figure S17–S19).

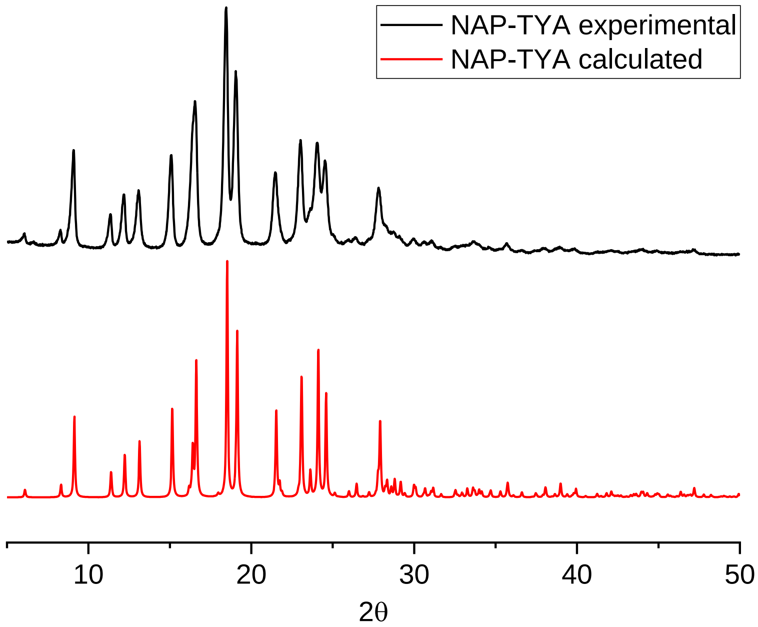

Figure S17. Experimental X-ray powder patterns of NAP-TYA (black, top) and simulated powder pattern calculated from the structure solved *via* SCXRD (red, bottom).

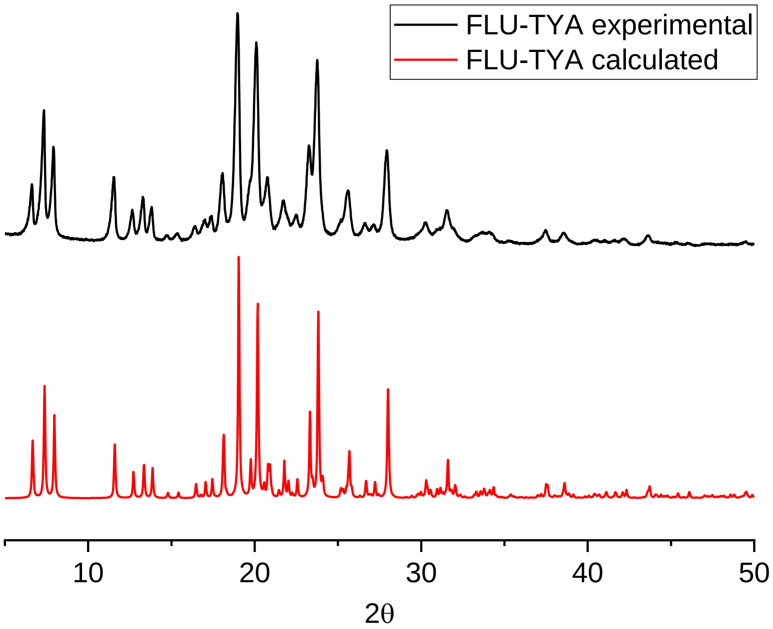

Figure S18. Experimental X-ray powder patterns of FLU-TYA (black, top) and simulated powder pattern calculated from the structure solved *via* SCXRD (red, bottom).

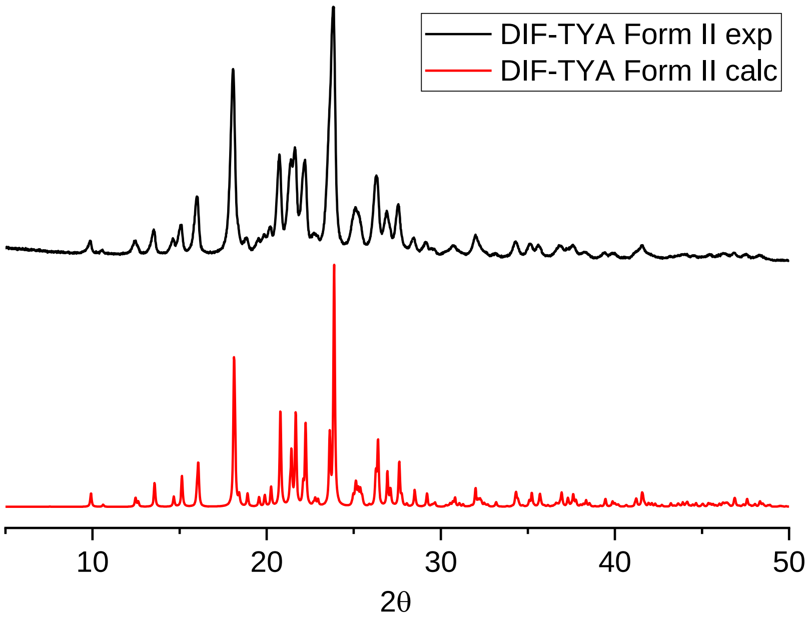

Figure S19. Experimental X-ray powder patterns of DIF-TYA (black, top) and simulated powder pattern calculated from the structure solved *via* SCXRD (red, bottom).

NAP-TYA and **FLU-TYA** crystallize into monoclinic space groups $P2_1$ and $P2_{1/n}$, respectively. In both cases the asymmetric unit consists of one independent molecule of API and one of **TYA**, indicating a stoichiometric API-coformer ratio of 1:1. In both structures, a $R_4^4(10)$ ring motif is established among two API and two TYA molecules through the following H-bonds: N1–H1A···O1 (N1···O1 = 2.760(5) Å), O2···H1C–N1 (O2···N1 = 2.759(6) Å), and N1–H1B···O2 (N1···O2 = 2.749(5) Å) for NAP-TYA (Figure S20a); N1–H1B···O2 (N1···O2 = 2.816(7) Å), O1···H1A–N1 (O1···N1 = 2.784(7) Å), and N1–H1C···O1 (N1···O1 = 2.811(7) Å) for FLU-TYA (Figure S21a). The heterotetramers are arranged in an alternate zig-zag tape (Figures S20b and S21b), and the different tapes are connected by TYA molecules through the formation of hydrogen bonds between the hydroxyl groups of TYA and the deprotonated carboxylic acid groups of NAP (O4···O1 = 2.664(6) Å) (Figure S20c) or FLU (O3···O2 = 2.744(6) Å) (Figure S21c). For FLU-TYA a 50:50 occupancy of the fluorine atom on the C8 and C6 sites was observed (Figure S21a).

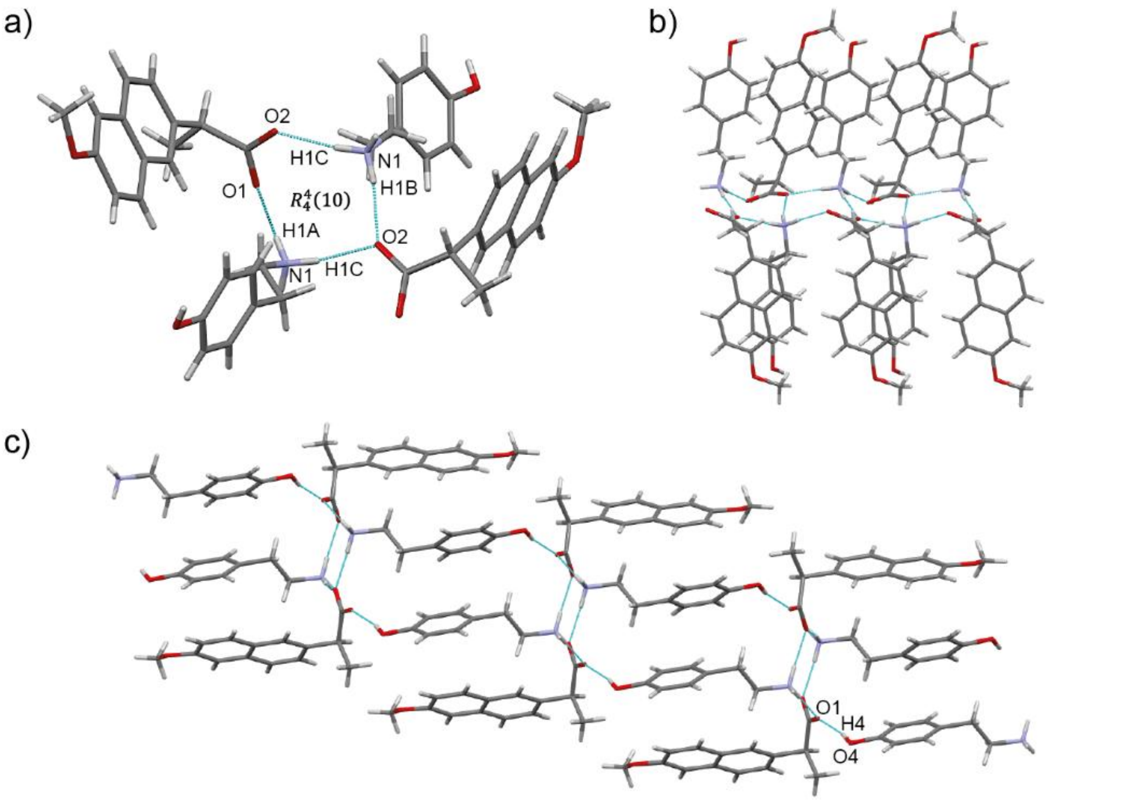

Figure S20. (a) $R_4^4(10)$ tetramer ring motif of NAP-TYA. (b) 1D chain formed by the linking of the $R_4^4(10)$ rings. (c) 2D hydrogen bonds network.

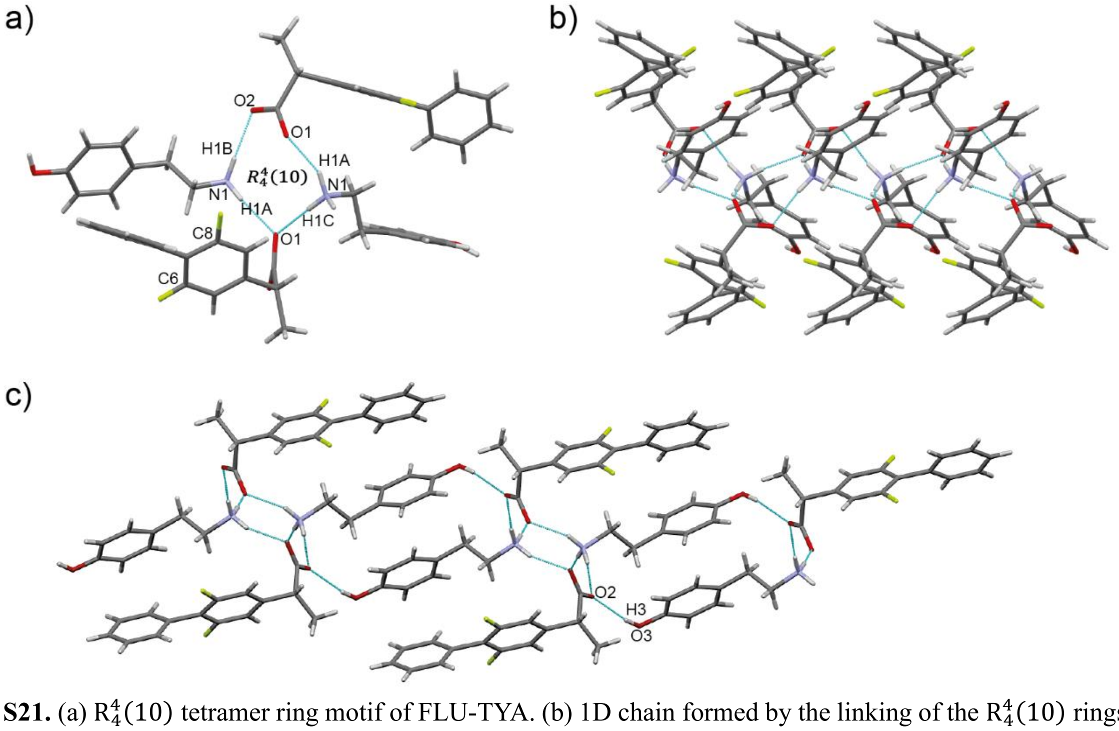

Figure S21. (a) $R_4^4(10)$ tetramer ring motif of FLU-TYA. (b) 1D chain formed by the linking of the $R_4^4(10)$ rings. (c) 2D hydrogen bonds network.

DIF-TYA Form II crystallizes into the orthorhombic space group *Pbca* in a 1:1 stoichiometric ratio. A 50:50 occupancy of the fluorine atom on the C9 and C13 sites was observed (Figure S22a). The intramolecular hydrogen bond (O1···O2 = 2.555(7) Å), already present in pure **DIF** and forming a $S(6)$ ring motif, is still observed (Figure S22a). Moreover, O2–H1B···N1 (O2···N1 = 3.326(6) Å), N1–H1A···O1 (N1···O1 = 3.023(6)

Å), O1–H1···O2 (O3···O1 = 2.555(6) Å), and N1–H1C···O3 (N1···O3 = 2.692(6) Å) H-bond interactions form a $R_5^5(12)$ ring motif involving four molecules. The tetrameric ring motifs are linked to each other and form 1D chains (Figure S22b). The different chains are then further connected by the involvement of the aromatic hydroxyl group of **TYA** in a hydrogen bond with the carboxylic group of **DIF** (O4···O2 = 2.652(2) Å). These interactions allow the establishment of a 2D hydrogen-bond network (Figure S22c), which is not observed in the structure of **DIF-TYA Form I**.[27]

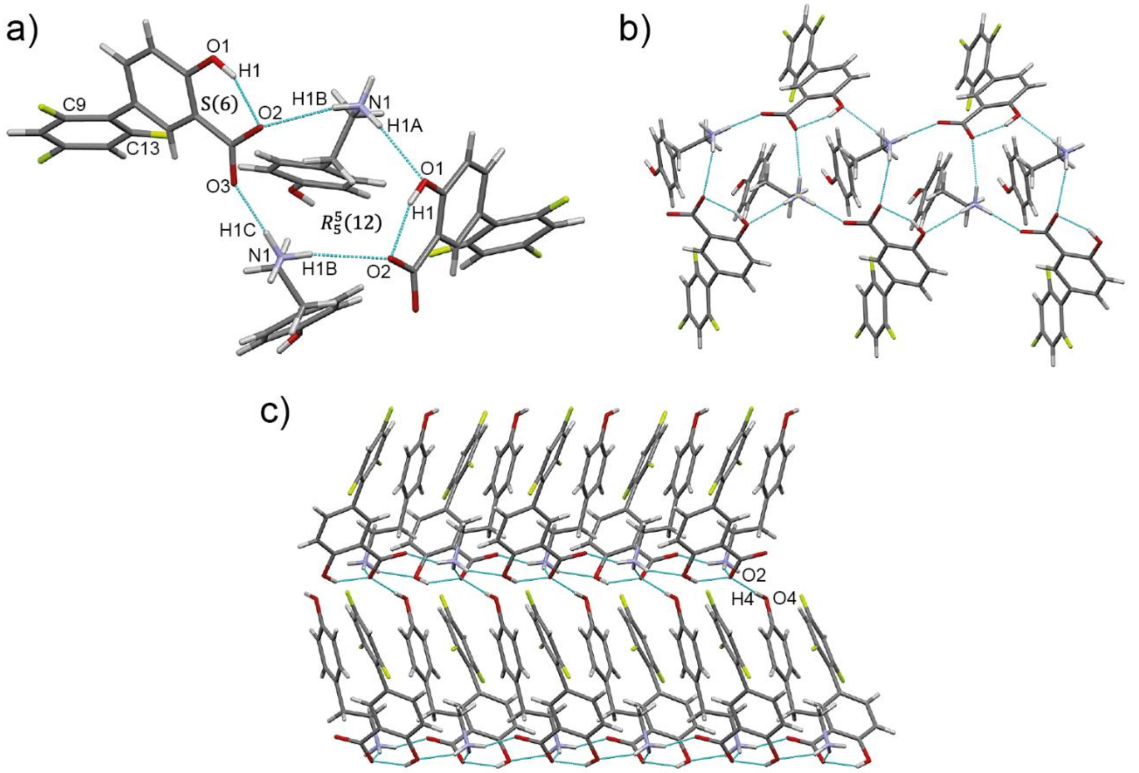

Figure S22. (a) S(6) and $R_5^5(12)$ ring motifs of DIF-TYA Form II. (b) 1D chain formed via N−H···O interactions. (c) 2D hydrogen bonds network.

4. NMR analysis

4.1 Comparison of the ^{13}C CPMAS spectra of the adducts and the starting materials

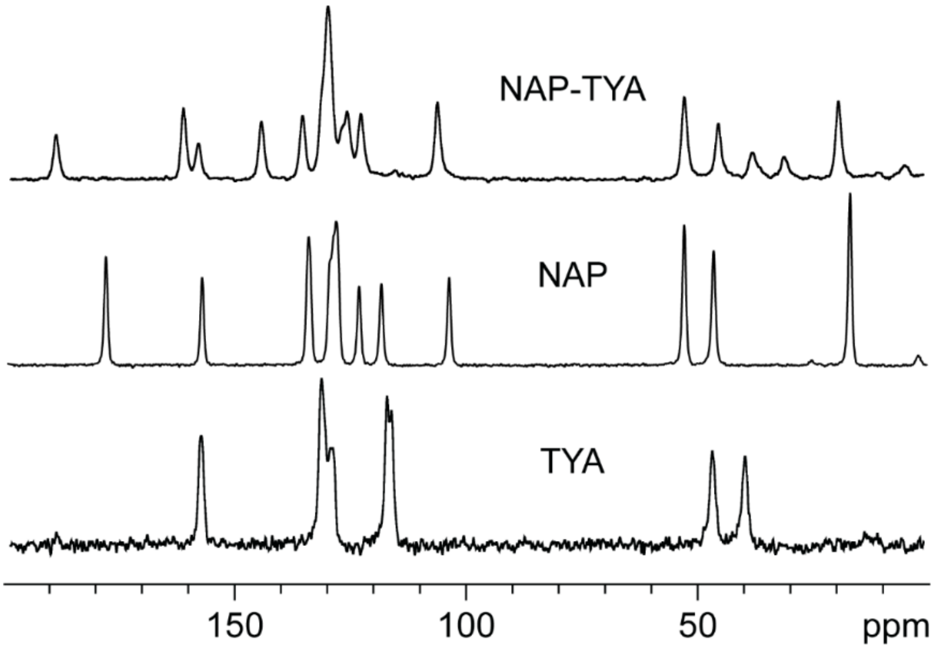

Figure S23. ^{13}C (100.63 and 150.91 MHz) CPMAS spectra of NAP-TYA (top), NAP (middle), and TYA (bottom) acquired at room temperature at a spinning speed of 12 and 20 kHz.

Table S2. ^{13}C resonances assignment of the starting materials NAP and TYA and the NAP-TYA adduct. The ^{13}C resonances exhibited by NAP were assigned based on previously reported data.[52] The atom numbering used is the one of the crystal structures COYRUD13 and SENJEC of NAP and TYA, respectively.

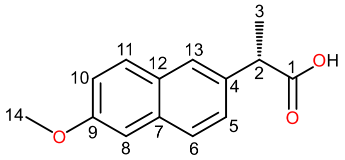

NAP

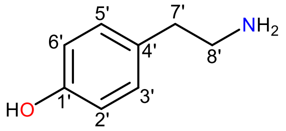

TYA

NAP-TYA

Carbon	δ (ppm)	Carbon	δ (ppm)	Carbon	δ (ppm)
C1	178.6	C1’	158.1	C1	184.7
C9	157.7	C4’	131.8	C9	158.5
C4/C7	134.5	C3’/C5’	129.8<br>129.3	C1’	155.4
C6/C11/<br>C12 /C13	129.8<br>129.1<br>128.5	C2’/C6’	117.4<br>116.4	C7/C12	142.4<br>133.9
C5	123.5	C8’	46.1	Aromatic C	129.8<br>128.7<br>125.7<br>124.7<br>121.8
C10	118.7	C7’	39.0	C8	106.0
C8	104.0			C14	55.0
C14	52.8			C2	47.9
C2	46.4			C8’	40.9
C3	16.8			C7’	34.3
				C3	23.1

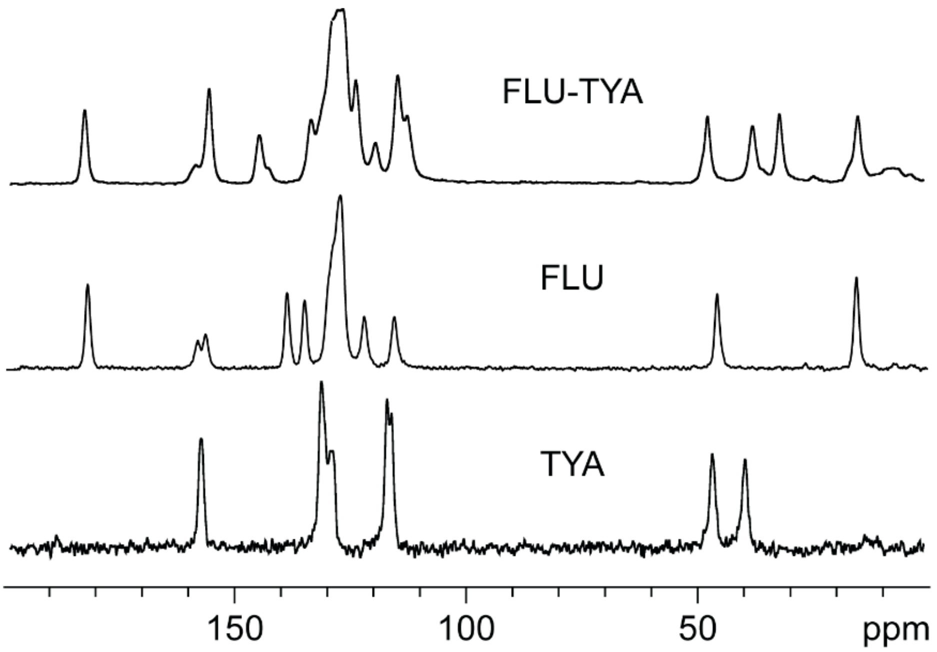

Figure S24. ^{13}C (100.63 and 150.91 MHz) CPMAS spectra of FLU-TYA (top), FLU (middle), and TYA (bottom) acquired at room temperature at a spinning speed of 12 and 20 kHz.

Table S3. ^{13}C resonances assignment of the starting materials FLU and TYA and the FLU-TYA adduct. The ^{13}C resonances exhibited by FLU were assigned based on previously reported data.[53] The atom numbering used is the one of the crystal structures FLUBIP and SENJEC of FLU and TYA, respectively.

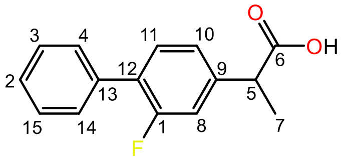

FLU

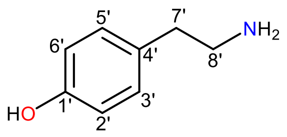

TYA

**FLU**		**TYA**		**FLU-TYA**	
**Carbon**	**δ (ppm)**	**Carbon**	**δ (ppm)**	**Carbon**	**δ (ppm)**
C6	183.4	C1’	158.1	C6	183.8
C1	159.4	C4’	131.8	C1/C1’	159.9
	157.7				157.0
C9	139.8	C3’/C5’	129.8	C9	146.3
			129.3		144.3
C13	136.0	C2’/C6’	117.4	C13	135.1
			116.4		
C2/C3/C4/	130.6	C8’	46.1		130.4
C11/C12/	129.8	C7’	39.0		129.4
C14/C15	128.2				128.3
C10	123.0			Aromatic C	125.5
C8	116.4				121.2
C5	45.9				116.5
C7	15.6				114.4
				C5	49.8
				C8’	40.1
				C7’	34.3
				C7	17.4

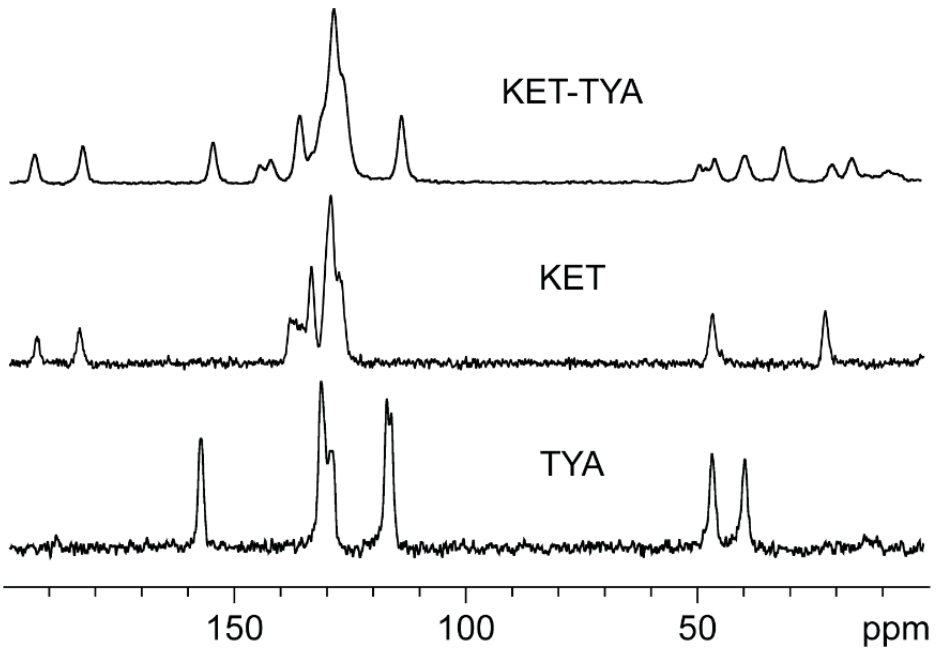

Figure S25. ^{13}C (100.63 and 150.91 MHz) CPMAS spectra of KET-TYA (top), KET (middle), and TYA (bottom) acquired at room temperature at a spinning speed of 12 and 20 kHz.

Table S4. ^{13}C resonances assignment of the starting materials KET and TYA and the KET-TYA adduct. The atom numbering used is the one of the crystal structures KEMRUP and SENJEC of NAP and TYA, respectively.

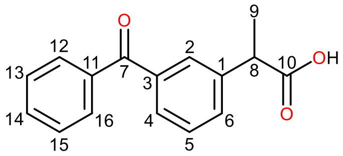 **KET**		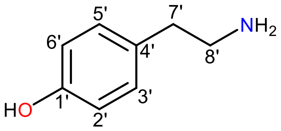 **TYA**		**KET-TYA**	
**Carbon**	**δ (ppm)**	**Carbon**	**δ (ppm)**	**Carbon**	**δ (ppm)**
C7	192.8	C1’	158.1	C7	194.3
C10	183.5	C4’	131.8	C10	184.0
C1/C3/C11	137.8	C3’/C5’	129.8	C1’	155.8
	135.2		129.3		
C6/C14	133.2	C2’/C6’	117.4		145.8
			116.4		
C2/C4/C5/	129.0	C8’	46.1		143.4
C12/C13/	127.2	C7’	39.0	Aromatic C	137.2
C15/C16					
C8	45.9				134.5
C9	21.5				132.0
					129.8
					128.1
				C2’/C6’	115.3
				C8	51.2
					48.0
				C8’	41.4
				C7’	33.1
				C9	22.6-18.4

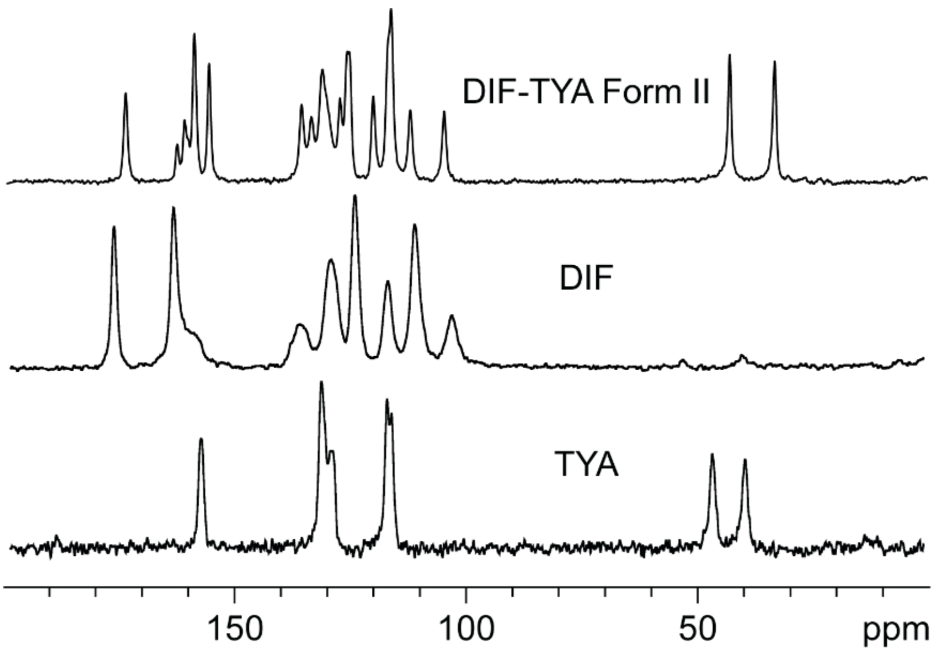

Figure S26. ^{13}C (100.63 and 150.91 MHz) CPMAS spectra of DIF-TYA Form II (top), DIF (middle), and TYA (bottom) acquired at room temperature at a spinning speed of 12 and 20 kHz.

Table S5. ^{13}C resonances assignment of the starting materials DIF and TYA and the DIF-TYA Form II adduct. The ^{13}C resonances exhibited by DIF were assigned based on previously reported data.[54,55] The atom numbering used is the one of the crystal structures FAFWIS01 and SENJEC of DIF and TYA, respectively.

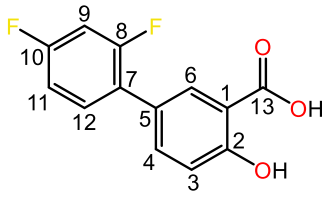 **DIF**		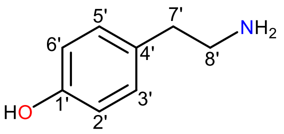 **TYA**		**DIF-TYA Form II**	
**Carbon**	**δ (ppm)**	**Carbon**	**δ (ppm)**	**Carbon**	**δ (ppm)**
C13	175.1	C1’	158.1	C13	173.8
C2	162.6	C4’	131.8	C2	162.6
C8/C10	158.5	C3’/C5’	129.8	C2/C8/C10/C1'	161.1
			129.3		160.2
C4/C6	136.0	C2’/C6’	117.4		158.9
			116.4		155.7
C12	129.5	C8’	46.1	Aromatic C	135.7
C5/C7	124.5	C7’	39.0		133.6
C3	117.6				131.1
C1/C11	112.0				127.3
C9	104.2				125.7
					125.3
					120.1
					116.3
				C1/C11	112.1
				C9	104.8
				C8’	42.8
				C7’	33.1

4.2 ^{1}H NMR spectrum of KET-TYA

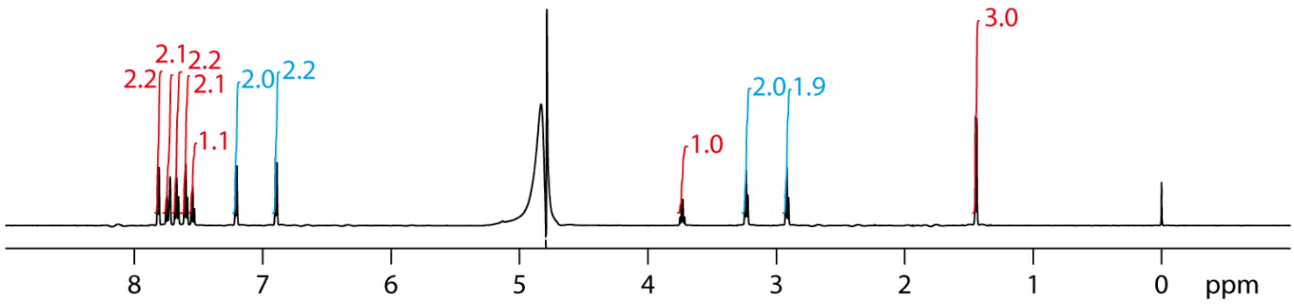

Figure S27. ^{1}H (600.17 MHz) NMR spectrum of KET-TYA acquired in water solution at 25 °C. The integrations of the TYA signals are shown in cyan, while in red the KET ones.

Table S6. ^{1}H signals (ppm) of the ^{1}H (600.17 MHz) NMR spectrum of KET-TYA.

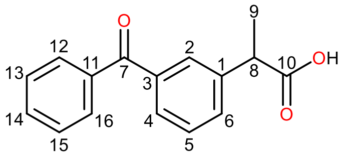

KET

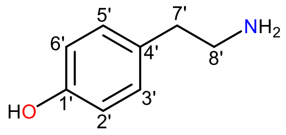

TYA

Signal	δ (ppm)	Signal	δ (ppm)
H12/H16	7.80	H3'/H5'	7.20
H2/H4	7.73	H2'/H6'	6.90
H6/H14	7.70	-C8'$\mathbf{H_2}$	3.23
H13/H15	7.60	-C7'$\mathbf{H_2}$	2.92
H5	7.54		
H8	3.72		
-C9$\mathbf{H_3}$	1.44		

5. Solubility tests

Table S7. Chemical shifts (δ, ppm) and integral values of the APIs' signals considered for the solubility tests in the solution ^1H NMR spectra. R1, R2, and R3 indicate the three measurements performed for each sample.

API	Signal	δ (ppm)	Integral			n° of protons
			R1	R2	R3	
NAP	CH_3	1.37	0.062	0.056	0.070	3
	-O–CH_3	3.80	0.064	0.061	0.070	3
FLU	CH_3	1.33	0.047	0.063	0.055	3
	H(Ar)	7.50	0.033	0.041	0.040	2
KET	CH_3	1.33	0.240	0.170	0.260	3
	H(Ar)	7.40	0.090	0.056	0.086	1
DIF	H(Ar)	7.60	0.028	0.026	0.030	1
	H(Ar)	7.90	0.028	0.026	0.029	1

Table S8. Chemical shifts (δ, ppm) and integral values of the adducts' signals considered for the solubility tests in the solution ^1H NMR spectra. R1, R2, and R3 indicate the three measurements performed for each sample.

Adduct	Signal	δ (ppm)	Integral			n° of protons
			R1	R2	R3	
NAP-TYA	CH_3	1.37	2.03	2.13	2.44	3
	-O–CH_3	3.80	2.03	2.12	2.48	3
FLU-TYA	CH_3	1.33	2.37	2.58	3.14	3
	H(Ar)	7.50	1.68	1.78	2.19	2
KET-TYA	CH_3	1.33	5.06	4.92	4.90	3
	H(Ar)	7.40	1.84	1.70	1.68	1
DIF-TYA Form II	H(Ar)	7.60	0.42	0.43	0.57	1
	H(Ar)	7.90	0.44	0.42	0.56	1

Table S9. Aqueous solubility values (mg/mL) obtained for APIs in pure form and in the synthesized adducts. The values were averaged for the three measurements made and are given with their standard deviation.

API	Solubility (mg/mL)	Adduct	Solubility (mg/mL)
NAP	0.021 ± 0.003	NAP-TYA	0.735 ± 0.075
FLU	0.019 ± 0.002	FLU-TYA	0.845 ± 0.042
KET	0.076 ± 0.017	KET-TYA	1.697 ± 0.058
DIF	0.028 ± 0.002	DIF-TYA Form II	0.473 ± 0.079

6. Experimental Section

Table S10. Amount of APIs and TYA (mmol, mg) used for the mechanochemical synthesis.

API	mmol	mg	Coformer	mmol	mg
NAP	0.217	50.0	TYA	0.217	29.8
FLU	0.205	50.0		0.205	28.1
KET	0.197	50.0		0.197	27.0
DIF	0.200	50.0		0.200	27.4

Table S11. Amounts of APIs in pure form and of the synthesized adducts introduced into vials with 4 mL of deionized H_2O for the solubility tests.

API	Amount (mg)	Adduct	Amount (mg)
S-naproxen	15.0	NAP-TYA	23.9
Flurbiprofen	15.0	FLU-TYA	23.4
Ketoprofen	15.0	KET-TYA	23.1
Diflunisal	15.0	DIF-TYA	23.2
